# Accessible hotspots for single-protein SERS in DNA-origami assembled gold nanorod dimers with tip-to-tip alignment

**DOI:** 10.1038/s41467-023-42943-7

**Published:** 2023-11-08

**Authors:** Francis Schuknecht, Karol Kołątaj, Michael Steinberger, Tim Liedl, Theobald Lohmueller

**Affiliations:** 1 Chair for Photonics and Optoelectronics, Nano-Institute Munich, Department of Physics, Ludwig-Maximilians-Universität (LMU), Königinstraße 10, 80539 Munich, Germany; 2 Physics Department and CeNS, Ludwig-Maximilians-University Munich, Geschwister-Scholl-Platz 1, 80539 Munich, Germany; 3 Present Address: Département de Physique, Université de Fribourg, Chemin du Musée 3, 1700 Fribourg, Switzerland

**Keywords:** Organizing materials with DNA, Nanoparticles, Raman spectroscopy

## Abstract

The label-free identification of individual proteins from liquid samples by surface-enhanced Raman scattering (SERS) spectroscopy is a highly desirable goal in biomedical diagnostics. However, the small Raman scattering cross-section of most (bio-)molecules requires a means to strongly amplify their Raman signal for successful measurement, especially for single molecules. This amplification can be achieved in a plasmonic hotspot that forms between two adjacent gold nanospheres. However, the small (≈1−2 nm) gaps typically required for single-molecule measurements are not accessible for most proteins. A useful strategy would thus involve dimer structures with gaps large enough to accommodate single proteins, whilst providing sufficient field enhancement for single-molecule SERS. Here, we report on using a DNA origami scaffold for tip-to-tip alignment of gold nanorods with an average gap size of 8 nm. The gaps are accessible to streptavidin and thrombin, which are captured at the plasmonic hotspot by specific anchoring sites on the origami template. The field enhancement achieved for the nanorod dimers is sufficient for single-protein SERS spectroscopy with sub-second integration times. This design for SERS probes composed of DNA origami with accessible hotspots promotes future use for single-molecule biodiagnostics in the near-infrared range.

## Abstract (web summary)

The identification of individual proteins is highly desirable in diagnostics. Here, the authors report on DNA-origami assembled dimers of gold nanorod with accessible hotspots to capture and identify single proteins from solution by SERS.

## Introduction

The label-free detection of single proteins or other small biomolecules from liquid samples is of great significance in biomedical diagnostics and pharmacology. Several experimental approaches have been developed towards this goal, such as nanopore conductance measurements^1^, interferometric detection^2^ and localized surface plasmon based sensing^3^. However, most methods do not provide detailed chemical information about the analyte, making an unambiguous identification challenging. Raman and infrared (IR) spectra on the other hand, provide a unique chemical fingerprint of the measured sample. While this seems ideal for label-free biomolecule detection, both methods also display limitations. IR spectroscopy is not compatible with measurements in aqueous solution due to the high absorption of water molecules in this wavelength range. Raman spectroscopy, on the other hand, is limited by the weak Raman scattering cross-sections of most molecules, which are typically between ≈10^−27^ to 10^−30^ cm^2^ ^4^. Additionally, large background noise and autofluorescence are often observed for biological samples, which renders single-molecule detection particularly challenging. Therefore, an enhancement of the Raman scattering intensity on the order of 10^7^ to 10^10^ is required for single-molecule (SM) Raman measurements^5,6^.

Surface-enhanced Raman scattering (SERS) exploits the product of the squares of the incident electromagnetic (EM) field enhancement and the polarizability enhancement at emission^7^. This is achieved by exposing molecules to EM hotspots generated by rough metal surfaces or plasmonic nanoantennas^8^. For example, tip-enhanced Raman spectroscopy has been applied to identify single proteins^9^. This technique exploits the high EM field enhancement at a sharp tip of a plasmonic probe to boost the Raman scattering intensity of analytes adsorbed on a substrate. Measuring spectra then requires scanning the sample with the probe. On a single-particle level, gold or silver nanostars feature sharp spikes, which provide a strong field enhancement sufficient for single-molecule detection, albeit with limited enhancement volume^10^. However, positioning an analyte precisely in the tip region can be challenging and sharp tips can display a limited stability^11^. A strong and highly confined EM field enhancement, by over two orders of magnitude, is further obtained in so-called plasmonic “hotspots” that occur due to plasmonic coupling between two nanoparticles forming a plasmonic dimer nanoantenna. In recent years, many examples of plasmonic dimers have been demonstrated as excellent probes for SM-SERS on dried samples^12^.

Optimizing the performance and applicability of plasmonic dimer nanoantennas towards SM-SERS involves several factors. Most importantly, the EM-field enhancement strength acts inversely to the interparticle distance. This limits the hotspot sizes to a few nm and requires accurate particle positioning. Furthermore, the analyte must be located precisely in the nanoparticle gap to benefit from the highest field enhancement. This second point is not an easy feat, particularly if one wants to add the analyte subsequently to preassembled dimers.

A highly successful approach for addressing both particle alignment and analyte positioning, is DNA self-assembly - the nanoscale folding of DNA into complex three-dimensional geometries^13,14^. The DNA origami method has been used to synthesize plasmonic dimer nanoantennas with controlled interparticle distances as highly reproducible and reliable SERS probes^15–22^. In particular, for example, SM-SERS was achieved with “nanofork”^21^ and “funnel”^18^ DNA origami designs, where the origami template hosting the analyte also spanned the interparticle gap. As a result, the plasmonic hotspots of these dimers were not freely accessible from the outside. Researchers have therefore devised DNA origami templates that displayed “open gaps”^15,17^. However, gaps in the range of ≈1–2 nm are typically required with nanosphere dimers to obtain sufficient signal enhancement for SM-SERS^12^. Such small hotspots cannot accommodate most proteins, which are a few nm in size^23^.

Dimer gaps large enough to accommodate single proteins while displaying a sufficient field enhancement for single-molecule detection have been obtained by switching from nanospheres to other particle shapes such as nanostars^24–26^, bipyramids^27^, and bowtie antennas^19^. For example, single-protein SERS was demonstrated by Tanwar et al., where thrombin was bound to a DNA template and then sandwiched between two bimetallic nanostars^28^. Heck et al.^22^ demonstrated SM-SERS on biotin/streptavidin with self-assembled “nanolenses” made of silver particles. Further, Zhan et al. reported non-resonant SERS of Cy5 in 5 nm wide gaps formed by DNA origami-assembled gold nanotriangles^19^.

However, most DNA origami-assembled dimer structures for SERS measurements are synthesized by following a similar protocol: A single dye or biomolecule is first attached to a DNA template, followed by the plasmonic nanoparticle assembly around the analyte. The resulting dimers are then typically purified by gel electrophoresis before conducting a SERS measurement. This synthetic procedure carries the advantage that most dimers are indeed labelled with the analyte, which is also positioned exactly in the plasmonic hotspot between the particles.

Arguably, a strategy of building the plasmonic dimer around the analyte is of limited use if one aims at identifying individual biomolecules such as proteins from a liquid sample. A reverse scheme based on capturing freely diffusing proteins from solution with a specific binding antagonist would be more desirable and applicable, as also pointed out by Tanwar et al.^28^. However, this requires accessible - i.e. large and open - hotspots for single proteins to enter via diffusion, while providing sufficient EM field enhancement over the protein volume at the same time. The design of plasmonic dimer nanoantennas with addressable binding sites thus requires finding a right balance between hotspot volume and Raman enhancement.

Here, we report on a DNA-origami design that enables the tip-to-tip alignment of gold nanorods (GNRs) with accessible interparticle gaps. GNRs display a larger plasmonic field enhancement for their longitudinal mode compared to gold nanospheres, due to an increase in tip curvature to volume ratio, and reduced surface plasmon damping^29^. The DNA origami-assembled gold nanorod dimers feature plasmonic hotspots, which are ≈8 nm wide and thus accessible for proteins of a smaller size from solution. This accessibility is demonstrated by SM-SERS detection of streptavidin and thrombin, where the analyte molecules enter the nanoantenna gaps during the SERS measurement procedure. We support the use of DNA origami-assembled nanorod dimers, as highly effective SERS probes, by comparing their calculated enhancement factors to those of well-established gold nanosphere dimers and other particle geometries.

## Results

### DNA origami design and assembly of GNR dimer nanoantennas

**Fig. 1:**
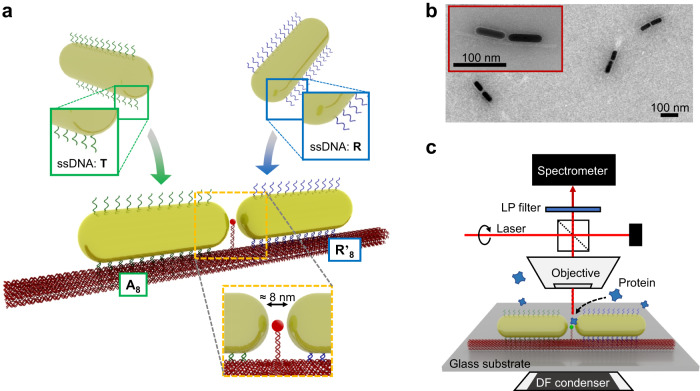
DNA origami design and dimer synthesis. **a** Gold nanorods are functionalised with different sequences of thiolated ss (single-strand) DNA (either T or R). Due to the differing labels, an individual nanorod can only bind to a designated binding site on the DNA origami support beam (either A_8_ or R’_8_) and nanorod dimers are formed. A docking site for the SERS analyte is precisely located between the nanorod tips, which form the plasmonic hotspot. **b** TEM image of GNR dimers assembled on the DNA origami support beam after sample purification. **c** Schematic of the SERS dark-field microscopy setup. Individual dimer nanoantennas are localized on a glass substrate by DFM. A 671 nm laser is coupled through the objective to perform SERS measurements on single nanoantennas, with analyte diffusing and binding into their hotspot gaps. A longpass (LP) filter is used to block the laser from entering the spectrometer.

The assembly of GNR dimer nanoantennas by DNA origami is illustrated in Fig. 1a. The DNA template was designed as a ≈215 nm long scaffold beam made of 14 DNA helices arranged in a honeycomb cross-section (Supplementary Fig. 1). The geometry of the origami was confirmed by transmission electron microscopy (TEM, Supplementary Fig. 2).

Each DNA origami beam exhibits two binding sites for attaching the GNRs. Each binding site is composed of a group of 14 DNA capture strands, which are spaced ~4 nm apart from one another. Both capture strands feature orthogonal sequences to prevent single nanorods from bridging the binding sites when binding to the template: an 8 nt long poly-A (A_8_), and a random (R’_8_) (ATGTAGGT) sequence.

The GNRs were synthesized with an aspect ratio (length:width) of ≈3, according to previously published protocols^30^. The average length of the nanorods was ≈63 nm (Supplementary Fig. 3), which provides a longitudinal plasmon mode within the bio-optical window for tissue, at around 690 nm (Supplementary Fig. 4). The synthesized nanorods were divided in two batches and functionalized with two different types of thiolated, single-stranded DNA (ssDNA), each complementary to one of the two binding sites on the DNA origami support beam. A small red-shift of the bulk GNR extinction spectrum by ≈2 nm was observed after ssDNA coating, indicative of a small increase of the effective external permittivity (Supplementary Fig. 4).

The GNRs were mixed with the DNA origami template in solution to form dimers. Prior to use, gel electrophoresis was performed to separate nanorod clusters and single nanorods from the target structures (Supplementary Fig. 5). The final GNRs dimers were analysed by TEM (Fig. 1b). From two synthesized nanorod batches, a nanogap size of 8.2 ± 2.6 nm and 8.6 ± 2.4 nm (tip-to-tip) was determined (Supplementary Fig. 6), with a nanorod dimer yield (with tip-to-tip orientation) of ≈55%. Subsequent SERS measurements on single dimers were conducted under a dark-field microscope (DFM, Fig. 1c).

### SM-SERS of Cy3.5 in water

**Fig. 2:**
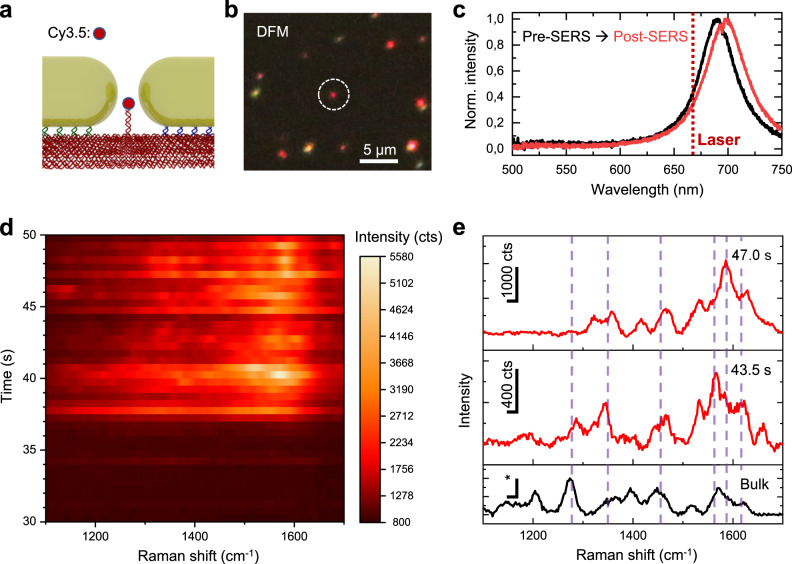
Non-resonant SERS from a single Cy3.5 molecule. **a** Sketch of the tip-to-tip alignment of GNRs with Cy3.5 at the nanoantenna gap. **b** Dark-field microscopy image of individual dimers on a glass substrate. Nanorod dimers are visible as red spots. **c** Scattering spectra of the GNR dimer circled in (**b**) before (black) and after (red) the SERS measurement. **d** Non-background subtracted SERS heatmap (intensity in counts: cts, integration time of 0.5 s) obtained from the dimer nanoantenna circled in b. **e** Single Cy3.5 Raman spectra (integration time of 0.5 s) from the measurement in d (at different points in time). Distinct Cy3.5 Raman peaks are marked with purple dashed lines (peak assignment provided in Table 1). The bulk spectrum was acquired from a 10 µM solution of Cy3.5 that was dried on a gold film that was sputtered onto a glass substrate. The scale bar marked by (*) of the bulk spectra corresponds to 4000 cts. Source data are provided as a Source Data file.

To determine the performance of the GNR dimer-origami structures for SM-SERS, samples hosting a single Cy3.5 molecule in the plasmonic hotspot between the nanorod tips were prepared (Fig. 2a). The measurement was conducted by drop-casting a solution of the purified nanoantennas onto a clean glass substrate. After a short incubation time (5-10 min), individual nanoantennas were settled on the glass surface and visible as bright, red spots in the DFM via their scattered light (Fig. 2b). Following this procedure, an average density of ca. 1000-1500 dimers per mm² was obtained (Supplementary Fig. 7). Furthermore, scattering spectra of individual structures were acquired to analyse the longitudinal plasmon resonance peak with respect to the Raman laser wavelength and to select suitable dimer nanoantennas for following SERS measurements (Fig. 2c). In principle, one can estimate the dimer gap size by reproducing the measured single dimer scattering spectrum with numerical finite difference time domain (FDTD) simulations (Supplementary Fig. 8). However, small deviations of the nanorod geometry or the dimer alignment can influence the result, which renders an unambiguous characterization without additional scanning electron microscopy (SEM) measurements challenging.

After having identified nanoantennas with DFM, SERS measurements were carried out in water with a focused 671 nm continuous wave (cw) laser at a power of 10 mW. The measurements were conducted using circularly polarized laser light to account for the random orientation of the nanorod dimers on the substrate. As shown in the heatmap displayed in Fig. 2d, the time-dependent SERS signal of the Cy3.5 displayed fluctuations, which is a common feature observed in SM measurements^31,32^. Two exemplary SERS spectra from the heatmap (obtained with integration times of 0.5 s) are shown in Fig. 2e. Characteristic Cy3.5 vibrational modes at ≈1270-1280 cm^−1^ (motions of aromatic groups)^33,34^, ≈1350 cm^−1^ (central methine chain motion)^33–35^, ≈1463 cm^−1^ (asymmetric CH_3_ deformation)^33–35^, 1560 and 1590 cm^–1^ (N^+^ = C stretching motion)^33,34,36^, and ≈1610-1620 cm^−1^ (C=C stretching mode)^34,36^ were identifiable (see also Table 1). The Cy3.5 SERS spectra also displayed good agreement with a reference spectrum of Cy3.5 obtained from a gold-coated glass substrate.

**Table 1:** Raman peak assignment

HD22/DNA Peaks (cm^−1^)	Assignment
1160-1170	DNA^42,47^
1230	Antisymmetric phosphate stretching^47^
1375	T, A, G ring breathing modes^42,47^
1490-1515	DNA & C^47^
1574	G, A^42,47^
1601	Thymine^42^
**Cy3.5 Peaks (cm**^**−1**^**)**	**Assignment**
1270-1280	Motion of aromatic groups^33,34^
1350	Methine chain motion^33–35^
1463	CH_3_ asymmetric deformation^33–35^
1560 & 1590	N^+^=C stretching motion^33,34,36^
1610 − 1620	C=C stretching mode^34,36^
**Biotin Peaks (cm**^**−1**^**)**	**Assignment**
1270	Methylene group wagging^43,44^
1470	Stretching of ring C-H_2_^43,44^
1565	C-N stretching^43,45^
**Streptavidin Peaks (cm**^**−1**^**)**	**Assignment**
1239	Amide III/β-sheet^43,44,46,47^
1336	Tryptophan W7^43,44,46^
1367	Tryptophan W7^43,44,46^, also DNA ring breathing^42,47^
1560-1580	Tryptophan W2^43,46^
1670	Amide I/β-sheet^43,46,47^
**Thrombin Peaks (cm**^**−1**^**)**	**Assignment**
1230-1250	Amide III/β-sheet^47,50^
1302−1310	Amide III or CH_2_ twisting in proteins or A, C^47^
1360	Tryptophan^47,50^
≈1550−1560	Amide II or tryptophan^47,50^
1639−1670	Amide I/β-sheet^47,50,51^

After the SERS measurement, a second dark-field scattering spectrum was taken to confirm that the nanorod dimer remained stable during the process. The post-SERS scattering spectrum looked almost identical in shape, although a small red-shift of the longitudinal plasmon peak by ≈8 nm was observed in this case (Fig. 2c). One potential source is attractive forces between the nanorods due to plasmon coupling induced by the focussed laser beam, which could pull the nanorods closer together, or a small contraction of the DNA origami template during the measurement. This in turn would increase the EM field enhancement, and could explain why Raman signals appeared only a few seconds after the start of the measurement. Strong plasmonic heating, which could destroy the dimer nanoantenna by particle melting or degradation of the DNA origami was avoided by performing the measurements in water, which has a significantly higher thermal conductivity than air. This argument is supported by the fact that dark-field scattering pre- and post-SERS spectra did not display a strong change. Additionally, a temperature increase would have led to dissociation of the analyte molecule connected to the DNA origami template by a single anchor strand^37^. Using lower laser powers for acquiring spectra is generally desirable, even in buffer, to minimize any temperature effects on the DNA origami, but can necessitate longer integration times. We therefore conducted protein measurements with different laser powers (0.5-5 mW) to balance both effects on the SERS signal.

### Single-protein SERS of streptavidin and thrombin

The applicability of the DNA origami GNR dimers for detecting proteins was determined by SM-SERS measurements of streptavidin (≈60 kg mol^−1^) and thrombin (≈36 kg mol^−1^). These proteins were chosen, as their molecular weight is close to the mean weight of proteins in eukaryotic cells (49 ± 48 kg mol^−1^)^38^. The tetrameric protein streptavidin has a diameter of ≈5 nm^39^, whilst thrombin has a hydrodynamic diameter of 4.1 nm^40^. The gaps of dimer nanoantennas were therefore large enough to accommodate a single protein.

To capture streptavidin from solution, the dimer hotspots were functionalized with a single-stranded biotin-labelled DNA anchor. The ability to define specific anchoring sites for single molecules on an origami scaffold is a major strength of DNA origami technology and has been demonstrated previously for many DNA origami designs to conduct single-molecule SERS^18,20,22,28^ and single-molecule localization microscopy^41^. In our case, the presence of a single anchoring site at the centre of the nanorod dimer, along with limited accessible space in the nanogaps ensured single-protein measurements.

**Fig. 3:**
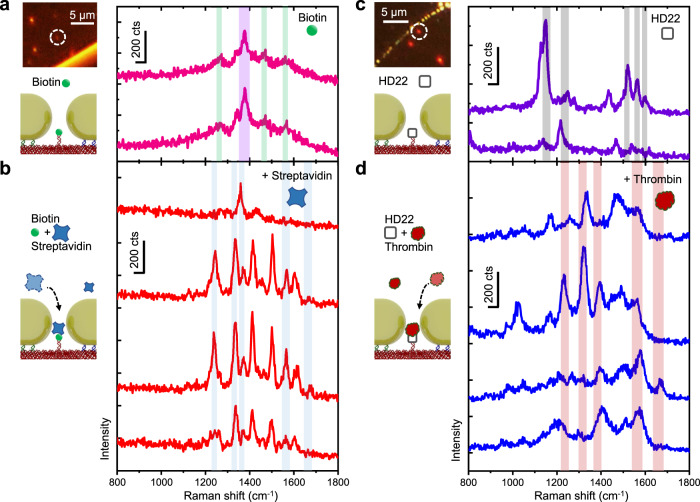
SERS spectra of single proteins. **a** DFM image of the corresponding dimer nanoantenna and sketch illustrating the nanogap filled with one biotin molecule, with example SERS spectra of only biotin (intensity in counts: cts). **b** Sketch illustrating the process of streptavidin entering the hotspot and binding to biotin, with SERS spectra obtained after adding streptavidin. The first spectrum in the stack was obtained shortly before streptavidin was captured by the antenna. Additional peaks corresponding to streptavidin are observed after the protein entered the nanogap (three bottom spectra). Raman peaks indicative of biotin and streptavidin are highlighted with green and blue bars respectively (see Table 1 for peak assignment). **c** DFM image of the corresponding dimer nanoantenna and sketch illustrating the nanogap filled with one aptamer, with SERS spectrum obtained from a dimer nanoantenna with HD22 before thrombin attachment. **d** Sketch illustrating the thrombin capturing process, with combined HD22/thrombin SERS spectra. All SERS spectra were obtained with 0.5 s integration times. Raman peaks indicative of HD22 or thrombin, are highlighted with grey and red bars respectively (see Table 1 for peak assignment). Source data are provided as a Source Data file.

The nanoantennas were drop-cast on a glass cover slip, and individual dimers were identified by DFM. The complete measurement comprised two steps. Firstly, SERS measurements of biotin-functionalized dimers were conducted in TE buffer with a laser power of 5 mW to confirm the presence of biotin in the hotspots and to determine any background Raman signal stemming from the DNA origami (Fig. 3a). The obtained spectra were dominated by a central double peak at ≈1375 cm^−1^, which most likely stems from nucleobases of the docking strand or origami template (ring breathing modes of T, A, and G bases)^42^. However, weaker Raman peaks indicative of biotin were also visible, at 1270 cm^−1^ (methylene group wagging)^43,44^, 1470 cm^−1^ (stretching of CH_2_)^43,44^, and 1565 cm^−1^ (C-N stretch)^43,45^_._

Next, streptavidin in TE buffer was added to the sample. After this, SERS spectra changed and additional peaks indicative for streptavidin binding in the nanoantenna gap appeared (Fig. 3b). The additional spectra displayed characteristic streptavidin Raman modes at 1239 cm^−1^ (amide III/β-sheet)^43,44,46,47^, 1336 cm^−1^ (tryptophan W7)^43,44,46^, 1560-1580 cm^−1^ (tryptophan W2)^43,46^ as well as 1670 cm^−1^ (amide I/ β-sheet)^43,46,47^ (Fig. 3b), which also appeared in bulk measurements of streptavidin (further examples of single dimer and bulk spectra are shown in Supplementary Fig. 9). Furthermore, a strong peak at ≈1503 cm^−1^ was featured in the spectrum, which is not specific to streptavidin and assignable to aromatic ring vibrations or *N* = H stretching^44,47^.

To demonstrate the binding specificity of streptavidin capturing by the dimer antennas, control measurements were conducted where the DNA origami dimers were incubated with myoglobin instead of streptavidin. Myoglobin was chosen to confirm specificity, as it is smaller than streptavidin (17 kg mol^−1^; hydrodynamic radius ≈ 1.75 nm)^48^ and does not bind to the biotin anchor. Even after prolonged measurements, we were not able to obtain a myoglobin SERS spectrum. In comparison, measurements where myoglobin and streptavidin were added at the same time, yielded a SERS spectrum matching the bulk Raman spectrum of streptavidin (Supplementary Fig. 10).

To further test the viability of our approach, we conducted a second experiment with thrombin, a globularly shaped enzyme involved in blood coagulation, which is slightly smaller than streptavidin^49^. As a binding antagonist, the anti-thrombin aptamer HD22 was used to label the anchor point of the DNA template. HD22 consists of 29 nucleotides that form a duplex/G-quadruplex mixed structure. From the SERS spectrum, the presence of the HD22 cannot be unambiguously confirmed, since any Raman peaks could also originate from the DNA origami itself. SERS measurements of the HD22 binding aptamer commenced in PBS buffer. Typical Raman modes for DNA were observed at 1160-1170 cm^−1^ (G^42,47^), 1375 cm^−1^ (T, A, and G ring breathing modes^42,47^), and 1574 cm^−1^ (G, A^42,47^). Additionally, a peak at 1230 cm^−1^ for antisymmetric phosphate stretching^47^ is assignable to origami, linker DNA or/and the HD22 aptamer (Fig. 3c).

Next, thrombin was added. The following measurement on single dimers was conducted with a laser power of 2 mW. Again, thrombin binding in hotspots was observable by SERS. Characteristic protein peaks for thrombin at 1230-1250 cm^−1^ (amide III/β-sheet^47,50^) as well as at 1360 cm^−1^ (tryptophan^47,50^), and ≈1550-1560 cm^−1^ (amide II range or tryptophan^47,50^) appeared in the SERS spectrum obtained from individual nanorod dimers. Notably, a characteristic amide I peak between 1639-1670 cm^−1^^47,50,51^ was not reliably observed for all spectra. This corresponds to findings, where the amide I vibrational mode can be suppressed in protein SERS and TERS measurements, by Kurouski et al.^52^ (Fig. 3d, additional dimer spectra and the thrombin bulk spectrum are shown in the Supplementary Fig. 9).

### Theoretical Raman enhancement of GNR dimers

**Fig. 4:**
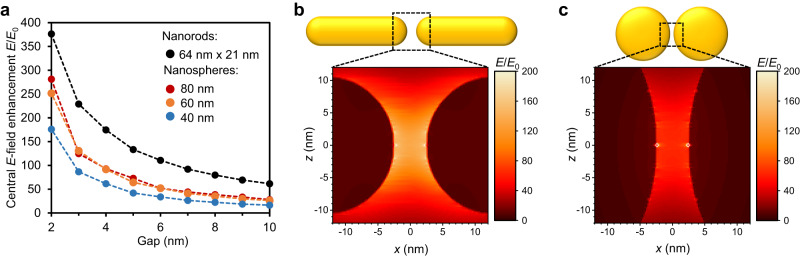
*E*-field enhancement for nanorod- compared to nanosphere dimers. **a** Comparison of the calculated maximum *E*-field enhancement (at corresponding resonance wavelengths) at the nanogap centre for different gold nanosphere- and gold nanorod dimers. **b** Calculated maximum *E*-field enhancement distribution between the tips of two nanorods (excited at 808 nm), assuming a tip-to-tip distance of 5 nm. **c** Calculated maximum *E*-field enhancement distribution between two 60 nm gold spheres separated by 5 nm (excited at 607 nm). All calculations were performed for nanostructures in water on a glass substrate at their hotspot centre resonance.

Based on the obtained SERS results, the question arises what field enhancement can be expected in the plasmonic hotspots of the nanorod dimers and whether single-molecule detection is feasible for this nanoantenna geometry. We performed FDTD calculations for the GNR dimer nanoantennas in water to determine the EM-field enhancement for different gap sizes, and to benchmark the performance of gold nanorod against nanosphere dimers. For these calculations, spherically end-capped nanorods with a width of 21 nm and a length of 64 nm were compared to gold nanospheres with diameters of 40, 60 and 80 nm. As shown in Fig. 4a, the maximum field enhancement for nanorod dimers exceeds the field enhancement of two nanospheres in the centre of the nanoantenna gap for all interparticle distances between 2 and 10 nm. Even for a 6-7 nm gap, the central |*E*|^4^ enhancement for nanorod dimers exceeds 10^8^. As an additional comparison, the field enhancement obtained in the centre of 5 nm gaps between nanorods is similar to that of nanospheres with a gap length of 3 nm. For the latter, non-resonant SM-SERS detection was reported in literature^21^. Further illustrating this point, the hotspot volume in which *E*/*E*_0_ (or enhancement factor EF) exceeds 100 (corresponding to an |*E*|^4^ enhancement of 10^8^) is on the order of 100 nm³ for nanorod dimers with a 5 nm large gap (approximately the size of a streptavidin or thrombin molecule), whilst similarly arranged 60 nm spheres feature an *E*/*E*_0_ of ≈ 50 (Fig. 4b, c). The latter is approximately the same maximum field enhancement one would already obtain at the tip of a single gold rod. We performed control measurements for streptavidin attached to biotin with a DNA origami structure hosting a single rod instead of a dimer (Supplementary Fig. 11). In this case, a protein SERS spectrum could not be obtained. This finding is supported by simulations, which show that the EF^4^ is ≈3 orders of magnitudes lower at monomer tips compared to dimer gaps.

Compared to gold spheres, nanorods are also advantageous for geometrical reasons. For the rods, even a short anchor strand that protrudes from the DNA origami template into the nanogap, was sufficient to localize the analyte molecule in the area of highest field enhancement. For 60 nm nanospheres, assembled in the same configuration, such a linker strand would have to be around as long as the sphere radius (30 nm). With a typical ≈50 nm persistence length of double stranded DNA^53^, the exact positioning of analyte in the nanoantenna hotspot^54^ would thus be less probable between spheres.

## Discussion

The SM-SERS detection of freely diffusing streptavidin (4.2 nm × 4.2 nm × 5.6 nm)^39^ and thrombin (4.5 nm × 4.5 nm × 5.0 nm)^49^ requires a minimum gap size of ≈5 nm to allow a single protein to enter the hotspot. Increasing the gap size is generally detrimental to the SERS enhancement, which makes the results presented here particularly compelling. In theory, the GNR dimer system exceeds a reported minimum EF^4^ requirements for SM-SERS of 10^7^, even for ≈8 nm wide gaps at their centre^6^. Also, there are other factors, including the general nature of proteins, which are beneficial for SERS measurements. For one, the proteins and their molecular subgroups examined here are relatively large. Bigger molecules tend to be more polarizable, and thus display larger Raman cross-sections^8^. Furthermore, the protein subgroups often feature repeatedly in the protein’s secondary structure, such as the amide β-sheet in streptavidin. The observed peaks in the SERS spectra are therefore not limited by individual molecular vibrations. Instead, the observed peaks are a superposition of the signal from all functional groups in the molecule that display the same Raman active modes. This argument is further supported by the fact that the hotspot volume between the nanorod tips is similar to the size of a whole protein (Fig. 4b).

As stated previously, spectra were obtained with reduced laser powers to avoid sample degradation. Calculations with the finite element method (FEM) for nanorods separated by an 8 nm gap indicate that dimer temperatures in water do not rise above ≈49 °C for a laser power of 5 mW (Supplementary Fig. 12). At these temperatures, the onset of protein denaturation cannot be entirely excluded, which along with a small degree of protein movement in the nanogap, can explain the observed SERS fluctuations. Raman spectral fluctuations could, however, also be indicative for carbonization of the analyte, particularly in SM-SERS^55^, due to heating (up to several 100 °C), or photochemical analyte transformations via hot-electrons^56^.

Carbonization is characterized by the emergence of broad carbon D-band at ≈1350 cm^−1^ and a stronger G-band at ≈1580 cm^−1^ that dominate the time-averaged spectrum. We did not observe carbon formation in our single-protein measurements (Supplementary Fig. 13). For one, Heck et al. have shown that gold is less prone to induce carbon formation compared to silver, possibly due to a favourable surface chemistry^57^. Additionally, Bjerneld et al. reported that carbonization is suppressed when SERS measurements are conducted in water^58^.

An additional important point to be considered is the probability for a single protein to actually diffuse into a nanogap. The mean diffusion time for single proteins (Supplementary Fig. 14) was approximated with a 3D random walk model. In general, the mean diffusion time is concentration dependent. For physiologically relevant concentrations of thrombin^59^ the estimated time for a protein to enter an available nanogap ranges from ≈2 h to ≈10 s respectively. For our measurements, a concentration range between 330 nM–4.2 µM (streptavidin) and 6.9–61 µM (thrombin) was investigated. Individual SERS spectra were acquired from dimer nanoantennas after incubation times of at least a few min, which excludes the mean diffusion time as a limiting factor. In fact, we did not observe any clear influence of analyte concentration in our experiments. For the single-dimer measurements reported here, other factors, such as the individual dimer geometry and the general orientation of the nanoantennas on the substrate play a more important role.

Averaged over all of our measurements, approximately ≈10-15% of DNA origami dimers delivered SERS. At first glance, this yield appears low. However, only dimers within a certain gap size range can be expected to deliver a single-protein spectrum, when they are both large enough to fit a single protein and small enough to provide sufficient field enhancement. Additionally, not all nanorod dimers on the substrate were aligned perfectly. An average alignment angle of 167° was determined by TEM measurements (Supplementary Fig. 6). The nanorod alignment itself does not alter the field enhancement in the nanoparticle gap significantly up to an angle of 150°, as shown by FDTD calculations (Supplementary Fig. 15). It could, however, lead to misalignment of the linker strand, and thus lower the accessibility of the binding site in the hotspot. Potential strategies to improve the SERS yield could include a further stabilization of the origami template, which could be achieved by DNA silanization^60^.

To verify that the gap size is indeed critical, we performed measurements with biotin-labelled dimer nanorod antennas on biotin-binding immunoglobulin G (IgG, 0.13 µM). IgG is larger than streptavidin and thrombin (≈150 kg mol^−1^; 14.5 nm × 8.5 nm × 4 nm^61^; hydrodynamic diameter ≈10.6 nm^62^) and might not fit those nanogaps small enough to deliver sufficient SERS. Indeed, we did not obtain any protein SERS spectrum of IgG, even after extended incubation times beyond 100 min. This also confirms that unspecific protein binding to the dimer antennas does not yield a SERS signal.

However, the IgG control measurement raises the question of how the antenna design could be optimized to obtain reliable SM-SERS of even larger molecules. In principle, beyond optimising the existing DNA-origami scaffold, two strategies can be envisioned: (i) one could use nanorods with sharper tips that provide a larger field enhancement, or, (ii) additional nanorods could be aligned in a trimer or tetramer structure to obtain larger openings for molecules to enter. We have performed a theoretical study comparing these scenarios (Supplementary Fig. 16). For sharper tips (at the gap), one finds that the EF is indeed higher close to the particle surface (similar to the tips of gold nanostars or -triangles) but decreases around the nanogap centre. Furthermore, sharp tips are generally more prone to melting and deformation upon laser excitation, even at low intensities. Blunter tips, on the other hand, provide a larger volume of more homogenous field enhancement, which is beneficial for proteins occupying almost the entire hotspot between the nanorods. As mentioned earlier, SERS of proteins benefits from a superposition of the SERS signal obtained from all Raman active vibrations of the molecule. Therefore, nanorods with spherical ends appear well suited to obtain a signal of the whole protein.

Adding more nanorods to form trimer and tetramer structure (Supplementary Fig. 16) can provide larger hotspots for analytes bound in the centre of the structure, and an additional axis of SERS enhancement (as dimers only enhance Raman along one spatial direction). However, for circularly polarised light, the strongest plasmonic coupling in this case is obtained between neighbouring nanorods. Central gaps formed by the trimer and tetramer antennas do - even when accounting for twice the field enhancement axes - not provide a stronger maximum Raman enhancement than similar dimers. However, for future studies of larger proteins (such as IgG), with more molecular subgroups, such designs might present a viable strategy.

Finally, practical factors in favour of nanorod dimers are considered. For one, the nanoantennas display plasmon resonances at wavelengths above 670 nm. This is an advantage, as the autofluorescence background of biological samples lies at lower wavelengths. Additionally, for SERS, the plasmonic nanoantennas operate fully within the near-infrared (NIR) or bio-optical window of tissue, where light has a high penetration depth. Since the nanoantennas are assembled and stabilized by the DNA origami in solution, they are freely deployable and could be injected as SERS probes into tissue and potentially operate in living cells. Combined with their addressability for specific biologically relevant proteins, future GNR dimer based in situ or in vivo measurements appear tangible. To realize such measurements, the nanosensors could be further protected by a layer of silica to preserve their integrity in cells. Importantly, site-specific silanization of DNA origami has already been demonstrated^63,64^, showing that it is indeed possible to selectively protect nanoantennas without hindering the binding of analyte molecules to be detected.

## Methods

### Chemicals

Ascorbic acid (99%), gold (III) chloride trihydrate (HAuCl_4_, 99%), hexadecyltrimethylammonium bromide (CTAB, ≥99%), magnesium chloride (MgCl_2_, ≥98%), sodium borohydride (NaBH_4_, ≥98%), hydrochloric acid (HCl, 37%), sodium dodecyl sulfate (SDS, 10% in H_2_O), as well as streptavidin (SKU 189730 and S4762), thrombin (SKU 1.12374), IgG (anti-biotin antibody produced in goat, SKU B3640) and myoglobin (from horse skeletal muscle; SKU M0630) were acquired from Sigma-Aldrich. Silver nitrate was purchased from TCI America (99%). All DNA strands were acquired from Eurofins Genomics and Biomers GmbH. Ultrapure water (resistivity: 18.2 MΩ cm at 25 °C) was obtained from a Milli-Q water purification system. All chemicals were used as received without further purification or treatment.

### DNA origami scaffold design and synthesis

The DNA origami structure was designed using caDNAno software^65,66^ A honeycomb lattice of 14 DNA strands (14 HB) with a total length of 215 nm was used. Two binding sites for nanorods were introduced along the surface of DNA origami. The gap between the nanorod binding sites featured the anchor strand for the analyte. Each binding site consisted of 14 staple strands extended by 8 nucleotides protruding out of the structure. The binding sites were extended with two different sequences, i.e. A_8_ (poly-A8 - AAAAAAAA) and R’_8_ (Random’ - ATGTAGGT). In the hotspot, two complementary DNA strands were introduced to elevate the Cy3.5 dye, the biotin molecule, and the HD22 aptamer above the DNA origami surface. The origami strand diagram and details on the hotspot design can be found in Supplementary Fig. 1. DNA sequences are given in Supplementary Table 1.

The folding of 14 HB DNA origami was carried out in TE buffer (Sigma-Aldrich) containing 10 nM of an 8634 nt scaffold, 100 nM DNA staples, 10 mM Tris (Sigma-Aldrich), 1 mM EDTA pH 8, and 24 mM MgCl_2_ (Sigma-Aldrich). Firstly, the mixture was heated up to 65 °C to ensure denaturation of all DNA strands and then cooled down to 4 °C in 19 h using a thermo cycler. The DNA origami structures were purified from an excess of DNA staples using filtration (100 kg mol^−1^ Amicon filters, 5 min, 10,000 × *g*). Filtration was repeated at least 5 times until no DNA signal was observed in the filtrate. To ensure the stability of the structures TE 6 mM MgCl_2_ buffer was added after each centrifugation step.

### Synthesis and functionalization of Au nanorods

Gold rods were synthesized using the seed-mediated method developed by Ming et al.^30^. The amount of silver ions during synthesis was varied to tune the nanorod aspect ratio. In the first stage of the synthesis, small gold nanoparticles (i.e., seeds) were formed by injecting a freshly-prepared, 4 °C NaBH_4_ solution (0.6 mL, 10 mM) into a rapidly stirred solution of HAuCl_4_ (0.25 mL, 10 mM) and CTAB (9.75 mL, 100 mM). Stirring was continued for 10 min. Subsequently, the solution was transferred to a thermostat and kept at 30 °C for 2 h. In the following step, obtained seeds were diluted 10x in a CTAB solution (0.1 mL seeds and 0.9 mL, 100 mM CTAB), and then added to a mixture of CTAB (100 mL, 0.1 M), HAuCl_4_ (5 mL, 10 mM), AgNO_3_ - 1 mL (for batch R1) or 0.75 mL (for batch R2) with 10 mM, HCl (2 mL, 1 M), and ascorbic acid (0.8 mL, 100 mM). After introducing the seed solution, the mixture was gently stirred for 10 s and then kept at 30 °C for 2 h. The solution turned red during this time, indicating the growth of gold rods. To remove any excess of reagents, the nanorod solutions were centrifuged two times for 10 min at 5000 × *g* and re-dispersed in a 0.2 mM CTAB solution. Before functionalization with DNA, the nanorods were once again centrifuged for 10 min at 5000 × g and re-suspended in 0.1% SDS solution. The dimensions (length and width) of the obtained gold nanorods were 63.4 nm x 20.5 nm (batch R1) and 62.7 nm x 24.0 nm (batch R2) (Supplementary Fig. 3).

The nanorods were labelled with two different 5’ thiolated DNA sequences to enable their binding to the DNA origami structures: poly-T19 and Random (TTCTCTACCACCTACAT). Both of them were complementary to DNA sequences of the binding sites on the DNA origami beam. Nanoparticle functionalization was carried out via a freeze and thaw method^67^. In this method, the growth of ice crystals during a freezing process increases the local concentration of both nanoparticles and DNA molecules, which enables a high labelling yield. Here, 300 µL, 0.1% SDS solution of 3 nM rods were mixed with 200 µL of 100 µM ssDNA to get a final ratio of GNR/DNA = 1/22,222. Afterwards, the solution was placed in −80 °C for 30 min, and then melted in an ultrasonic bath. Functionalized nanorods were purified from an excess of DNA strands using electrophoresis (1% agarose, 70 V, 2 h in 1 x TAE, 11 mM MgCl_2_ buffer).

### Binding of nanoparticles to DNA origami

The synthesis of nanorod dimers on DNA origami was realized through hybridization between DNA-functionalized nanorods and complementary strands on a DNA origami surface. Firstly, 20 µL of 20 nM rods solution was added to 20 µL of 2 nM 14 HB under vigorous stirring. Then, the solution was annealed from 45 °C to 20 °C for 20 h. Finally, the obtained dimers were purified from an excess of unbound nanorods by gel electrophoresis (1% agarose, 70 V, 2 h in 1 x TAE, 11 mM MgCl_2_ buffer).

### TEM measurements

For TEM imaging, 3 µL of sample solution were dropped onto formvar-coated grids (300 mesh Cu, Ted Pella Inc). After 1 min the solution was removed with a paper filter, and the sample was negatively stained for 10 s using 2% Uranyl formate solution. All TEM measurements were carried out using a JEM-1101 electron microscope (JEOL) with an accelerating voltage of 80 kV.

### Deposition of GNR dimers on glass slides

For SERS, dark-field, and SEM measurements obtained dimers were deposited on the surface of 170 µm thick glass slides (Carl Roth GmbH, Germany). Firstly, glass slides were thoroughly cleaned by bath sonication in Hellmanex III (Sigma-Aldrich), acetone, isopropanol, and in Milli-Q water for 15 min each. Afterwards, glass slides were plasma cleaned in oxygen atmosphere for 5 min. Finally, 50 µL of diluted dimers solution was deposited on the surface for 1 min, followed by rinsing in distilled water and blow-dried with nitrogen.

### Optical microscopy and spectroscopy

Dark-field microscopy and spectroscopy as well as SERS measurements were carried out with a Zeiss Axio Scope A1. For dark-field illumination a Zeiss oil condenser (NA 1.2–1.4), and an unpolarised halogen white light source were used. A Canon EOS 6D was used for image acquisition. Fluid levels were kept at ≈100 μL, by injecting deionised water, to compensate for evaporation during the measurements. A Zeiss Achroplan 100x NA 1.0 objective was used for SERS measurements in water. Dark-field scattering images in air were taken with a Zeiss Epiplan 50x/NA 0.7 objective. Spectra were taken with a Princeton Instruments Acton SP2500 grating, coupled to a Princeton eXcelon CCD detector. A 671 nm Laser (500 mW model, Laser Quantum) was used for SERS measurements. Background subtraction was employed for the Raman spectra in Fig. 2e (Cy3.5), as well as those in Supplementary Figs. 8 and 10b (except for: Myoglobin only), for improved visual clarity.

### Numerical simulations

3D random walk modelling was used to estimate the concentration dependent mean diffusion time (MDT) of a protein for entering a nanogap (Supplementary Fig. 14). For the model, a fixed step-size of 4 nm, in a 400 nm x 400 nm x 400 nm large simulation box with reflective surfaces, and a freestanding 4 nm × 4 nm × 4 nm target “hitbox” was assumed. Different numbers of proteins were placed randomly in different locations of the simulation box. The simulations were run 100 times each. Step-times were approximated with the diffusion time of the 4 nm step-size. For this, diffusion coefficients $D$ of streptavidin (7.72 · 10^−11^ m² s^−1^) and thrombin (1.06 · 10^−10^ m² s^−1^) were calculated using the diffusion equation

1 $$D=\frac{{{{{{{\rm{k}}}}}}}_{{{{{{\rm{B}}}}}}}\cdot T}{6\pi \cdot r\cdot \eta }$$

Here, hydrodynamic radii $r$ of 2.82 nm for streptavidin^62^ and 2.05 nm for thrombin^40^ were used. To determine the MDTs, step-times were multiplied with average step-counts until a particle landed in the hitbox. Mean diffusion times are inversely related to protein concentration [protein], with MDT_S._ = 8.4 s µM [streptavidin]^−1^ for streptavidin, and with MDT_T._ = 6.1 s µM [thrombin]^−1^ for thrombin.

FDTD calculations were done with Lumerical (2020a FDTD Solver Version 8.23.2194, and newer). Meshing for 21 nm × 64 nm GNR, as well as 40 and 60 nm sphere-based dimers was set to 0.2 nm. For 80 nm sphere dimers, as well as the trimer and tetramer meshing was set to 0.4 nm. The mesh-volume embedding the particle was sized with an extra margin of ≈10% per spatial dimension. The overall simulation region was 4 µm x 4 µm × 4 µm in size. For dimers, min boundary conditions were set to asymmetric along, and symmetric perpendicularly to the polarization axis and the poynting vector of the linearly illuminating TFSF plane wave source. The light source was polarised along the dimers’ long axes, directed perpendicularly to the substrate plane (Supplementary Fig. 8). For the trimer and tetramer structures, two 90° phase and polarization shifted TFSF sources were used to account for additional geometric symmetries (here no symmetric boundary conditions were employed). Field enhancement factors were gained from hotspot centres, and renormalized with field strengths at an empty substrate under similar illumination conditions. The simulations were run until 10^−5^ as a fraction of the initial energy remained in the system. Material parameters for glass^68^, water^68^, and single-crystalline gold^69^ were taken from literature.

Heating of GNR dimers was modelled using COMSOL Multiphysics (version 5.2a). Meshing was set to be physics-controlled and extra fine. The simulation volume was 1 µm x 1 µm x 1 µm large, half water, half glass, with a boundary surface temperature of 20 °C. Material parameters were derived from the database of the software.

### Reporting summary

Further information on research design is available in the Nature Portfolio Reporting Summary linked to this article.

### Supplementary information

Supplementary Information

Reporting Summary

### Source data

Source Data

## Data Availability

The data that support the findings of this study are available from the corresponding authors upon request. Source data are provided with this paper.

## Code availability

Python code used to implement the random walk model for estimating hotspot diffusion times is available from the authors upon request.

## References

[1] Gu LQ, Shim JW (2010). Single molecule sensing by nanopores and nanopore devices. Analyst.

[2] Piliarik M, Sandoghdar V (2014). Direct optical sensing of single unlabelled proteins and super-resolution imaging of their binding sites. Nat. Commun..

[3] Zhang PF (2020). Plasmonic scattering imaging of single proteins and binding kinetics. Nat. Methods.

[4] Le Ru EC, Etchegoin PG (2012). Single-molecule surface-enhanced Raman spectroscopy. Annu. Rev. Phys. Chem..

[5] Blackie EJ, Le Ru EC, Etchegoin PG (2009). Single-molecule surface-enhanced Raman spectroscopy of nonresonant molecules. J. Am. Chem. Soc..

[6] Le RuEC, Blackie E, Meyer M, Etchegoin PG (2007). Surface enhanced Raman scattering enhancement factors: a comprehensive study. J. Phys. Chem. C..

[7] Ding SY, You EM, Tian ZQ, Moskovits M (2017). Electromagnetic theories of surface-enhanced Raman spectroscopy. Chem. Soc. Rev..

[8] Le Ru, E. C. & Etchegoin, P. G. *Principles of Surface-Enhanced Raman Spectroscopy and Related Plasmonic Effects* 1st edn, Vol. 1 (Elsevier, 2009).

[9] Kumar N, Weckhuysen BM, Wain AJ, Pollard AJ (2019). Nanoscale chemical imaging using tip-enhanced Raman spectroscopy. Nat. Protoc..

[10] Chatterjee, S. et al. A high-yield, one-step synthesis of surfactant-free gold nanostars and numerical study for single-molecule SERS application. *J. Nanopart. Res.* **18**, 242 (2016).

[11] Vega, M. M. et al. Long-term stability of surfactant-free gold nanostars. *J. Nanopart. Res.* **16**, 2729 (2014).

[12] Langer J (2020). Present and future of surface-enhanced Raman scattering. ACS Nano..

[13] Rothemund PWK (2006). Folding DNA to create nanoscale shapes and patterns. Nature.

[14] Douglas SM (2009). Self-assembly of DNA into nanoscale three-dimensional shapes. Nature.

[15] Pilo-Pais M, Watson A, Demers S, LaBean TH, Finkelstein G (2014). Surface-enhanced Raman scattering plasmonic enhancement using DNA origami-based complex metallic nanostructures. Nano. Lett..

[16] Kühler P (2014). Plasmonic DNA-origami nanoantennas for surface-enhanced Raman spectroscopy. Nano. Lett..

[17] Thacker, V. V. et al. DNA origami based assembly of gold nanoparticle dimers for surface-enhanced Raman scattering. *Nat. Commun.* **5**, 3448 (2014). 10.1038/ncomms4448 24622339

[18] Simoncelli S (2016). Quantitative single-molecule surface enhanced Raman scattering by optothermal tuning of DNA origami-assembled plasmonic nanoantennas. ACS Nano..

[19] Zhan PF (2018). DNA origami directed assembly of gold bowtie nanoantennas for single-molecule surface-enhanced Raman scattering. Angew. Chem. Int Ed..

[20] Fang WN (2019). Quantizing single-molecule surface-enhanced Raman scattering with DNA origami metamolecules. Sci. Adv..

[21] Tapio K (2021). A versatile DNA origami-based plasmonic nanoantenna for label-free single-molecule surface-enhanced Raman spectroscopy. ACS Nano..

[22] Heck C, Kanehira Y, Kneipp J, Bald I (2018). Placement of single proteins within the SERS hot spots of self-assembled silver nanolenses. Angew. Chem. Int. Ed..

[23] La Verde V, Dominici P, Astegno A (2017). Determination of hydrodynamic radius of proteins by size exclusion chromatography. Bio. Protoc..

[24] Tanwar S, Haldar KK, Sen T (2017). DNA origami directed Au nanostar dimers for single-molecule surface-enhanced Raman scattering. J. Am. Chem. Soc..

[25] Kaur V, Tanwar S, Kaur G, Sen T (2021). DNA-origami-based assembly of Au@Ag nanostar dimer nanoantennas for label-free sensing of pyocyanin. Chem. Phys. Chem..

[26] Kaur V, Sharma M, Sen T (2021). DNA origami-templated bimetallic nanostar assemblies for ultra-sensitive detection of dopamine. Front. Chem..

[27] Kaur C, Kaur V, Rai S, Sharma M, Sen T (2023). Selective recognition of the amyloid marker single thioflavin T using DNA origami-based gold nanobipyramid nanoantennas. Nanoscale.

[28] Tanwar S, Kaur V, Kaur G, Sen T (2021). Broadband SERS enhancement by DNA origami assembled bimetallic nanoantennas with label-free single protein sensing. J. Phys. Chem. Lett..

[29] Sonnichsen C (2002). Drastic reduction of plasmon damping in gold nanorods. Phys. Rev. Lett..

[30] Ming T (2009). Growth of tetrahexahedral gold nanocrystals with high-indexfacets. J. Am. Chem. Soc..

[31] Etchegoin PG, Le Ru EC (2010). Resolving single molecules in surface-enhanced Raman scattering within the inhomogeneous broadening of Raman peaks. Anal. Chem..

[32] Weiss A, Haran G (2001). Time-dependent single-molecule Raman scattering as a probe of surface dynamics. J. Phys. Chem. B.

[33] Sato H, Kawasaki M, Kasatani K, Katsumata MA (1988). Raman-spectra of some indo-carbocyanine, thia-carbocyanine and selena-carbocyanine dyes. J. Raman Spectrosc..

[34] Cao YWC, Jin RC, Mirkin CA (2002). Nanoparticles with Raman spectroscopic fingerprints for DNA and RNA detection. Science.

[35] Yang JP, Callender RH (1985). The resonance Raman-spectra of some cyanine dyes. J. Raman Spectrosc..

[36] Lednev IK (1992). A Raman-spectroscopic study of indolinium steryl dyes. Spectrochim. Acta. A.

[37] Jungmann R (2010). Single-molecule kinetics and super-resolution microscopy by fluorescence imaging of transient binding on DNA origami. Nano. Lett..

[38] Kozlowski LP (2017). Proteome-pI: proteome isoelectric point database. Nucleic Acids Res..

[39] van Oss CJ (2003). Macroscopic-scale surface properties of streptavidin and their influence on aspecific interactions between streptavidin and dissolved biopolymers. Colloid Surf. B.

[40] Kim OV, Xu ZL, Rosen ED, Alber MS (2013). Fibrin networks regulate protein transport during thrombus development. PLoS Comput. Biol..

[41] Steinhauer C, Jungmann R, Sobey TL, Simmel FC, Tinnefeld P (2009). DNA origami as a nanoscopic ruler for super-resolution microscopy. Angew. Chem. Int. Ed..

[42] Wu TC, Vasudev M, Dutta M, Stroscio MA (2013). Raman and Surface-Enhanced Raman Scattering (SERS) studies of the thrombin-binding aptamer. IEEE T Nanobiosci..

[43] Galarreta BC, Norton PR, Lagugne-Labarthet F (2011). SERS Detection of streptavidin/biotin monolayer assemblies. Langmuir.

[44] Rutherford G (2015). Photochemical growth of highly densely packed gold nanoparticle films for biomedical diagnostics. ECS J. Solid State Sci..

[45] Wang H, Schultz ZD (2013). The chemical origin of enhanced signals from tip-enhanced Raman detection of functionalized nanoparticles. Analyst.

[46] Fagnano C, Torreggiani A, Fini G (1996). Raman spectroscopic studies of the anhydrous complexes of avidin and streptavidin with biotin. Biospectroscopy.

[47] Movasaghi Z, Rehman S, Rehman IU (2007). Raman spectroscopy of biological tissues. Appl Spectrosc. Rev..

[48] Papadopoulos S, Jurgens KD, Gros G (2000). Protein diffusion in living skeletal muscle fibers: dependence on protein size, fiber type, and contraction. Biophys. J..

[49] Weisel JW, Nagaswami C, Young TA, Light DR (1996). The shape of thrombomodulin and interactions with thrombin as determined by electron microscopy. J. Biol. Chem..

[50] Rygula A (2013). Raman spectroscopy of proteins: a review. J. Raman Spectrosc..

[51] Pagba CV, Lane SM, Cho HS, Wachsmann-Hogiu S (2010). Direct detection of aptamer-thrombin binding via surface-enhanced Raman spectroscopy. J. Biomed. Opt..

[52] Kurouski D, Postiglione T, Deckert-Gaudig T, Deckert V, Lednev IK (2013). Amide I vibrational mode suppression in surface (SERS) and tip (TERS) enhanced Raman spectra of protein specimens. Analyst.

[53] Benson E (2018). Effects of design choices on the stiffness of wireframe DNA origami structures. ACS Nano..

[54] Chikkaraddy R (2018). Mapping nanoscale hotspots with single-molecule emitters assembled into plasmonic nanocavities using DNA origami. Nano. Lett..

[55] Otto A (2002). What is observed in single molecule SERS, and why?. J. Raman Spectrosc..

[56] Szczerbinski J, Gyr L, Kaeslin J, Zenobi R (2018). Plasmon-driven photocatalysis leads to products known from E-beam and X-ray-induced surface chemistry. Nano. Lett..

[57] Heck C, Kanehira Y, Kneipp J, Bald I (2019). Amorphous carbon generation as a photocatalytic reaction on DNA-assembled gold and silver nanostructures. Molecules.

[58] Bjerneld EJ, Svedberg F, Johansson P, Kall M (2004). Direct observation of heterogeneous photochemistry on aggregated Ag nanocrystals using Raman spectroscopy: The case of photoinduced degradation of aromatic amino acids. J. Phys. Chem. A.

[59] Wolberg AS, Campbell RA (2008). Thrombin generation, fibrin clot formation and hemostasis. Transfus. Apher. Sci..

[60] Nguyen L, Doblinger M, Liedl T, Heuer-Jungemann A (2019). DNA-origami-templated silica growth by sol-gel chemistry. Angew. Chem. Int. Ed..

[61] Tan YH (2008). A nanoengineering approach for investigation and regulation of protein immobilization. ACS Nano..

[62] Yusko EC (2017). Real-time shape approximation and fingerprinting of single proteins using a nanopore. Nat. Nanotechnol..

[63] Shang YX (2020). Site-specific synthesis of silica nanostructures on DNA origami templates. Adv. Mater..

[64] Wassermann LM, Scheckenbach M, Baptist AV, Glembockyte V, Heuer-Jungemann A (2023). Full site-specific addressability in DNA origami-templated silica nanostructures. Adv. Mater..

[65] Douglas SM (2009). Rapid prototyping of 3D DNA-origami shapes with caDNAno. Nucleic Acids Res..

[66] Hong F, Zhang F, Liu Y, Yan H (2017). DNA origami: scaffolds for creating higher order structures. Chem. Rev..

[67] Liu BW, Liu JW (2017). Freezing directed construction of bio/nano interfaces: reagentless conjugation, denser spherical nucleic acids, and better nanoflares. J. Am. Chem. Soc..

[68] Palik E. D. *Handbook of Optical Constants of Solids II* 1st edn, Vol. 2 (Academic, 1991).

[69] Olmon R. L., et al. Optical dielectric function of gold. *Phys. Rev. B* **86**, 235147 (2012).

