# Supplementary material for: Accessible hotspots for single-protein SERS in DNA-origami assembled gold nanorod dimers with tip-to-tip alignment
Source: Nat Commun. 2023 Nov 8;14:7192. doi: 10.1038/s41467-023-42943-7 (PMC10632510; doi:10.1038/s41467-023-42943-7)
Supplement: Supplementary file 1 — Supplementary Information [file 41467_2023_42943_MOESM1_ESM.pdf]

Supplementary Information for

Accessible hotspots for single-protein SERS in DNA-origami assembled gold nanorod dimers with tip-to-tip alignment

Francis Schuknecht[1], Karol Kołątaj[2, 3], Michael Steinberger[1], Tim Liedl[2]*, Theobald Lohmueller[1]*

[1]Chair for Photonics and Optoelectronics, Nano-Institute Munich, Department of Physics, Ludwig-Maximilians-Universität (LMU), Königinstraße 10, 80539 Munich, Germany

[2]Physics Department and CeNS, Ludwig-Maximilians-University Munich, Geschwister-Scholl-Platz 1, 80539 Munich, Germany

[3]Current Address: Département de Physique, Université de Fribourg, Chemin du Musée 3, 1700, Fribourg, Switzerland

These authors contributed equally: Francis Schuknecht, Karol Kołątaj

*Corresponding authors: tim.liedl@physik.uni-muenchen.de (T.Li.); t.lohmueller@lmu.de (T.Lo.)

Supplementary Figure 1:

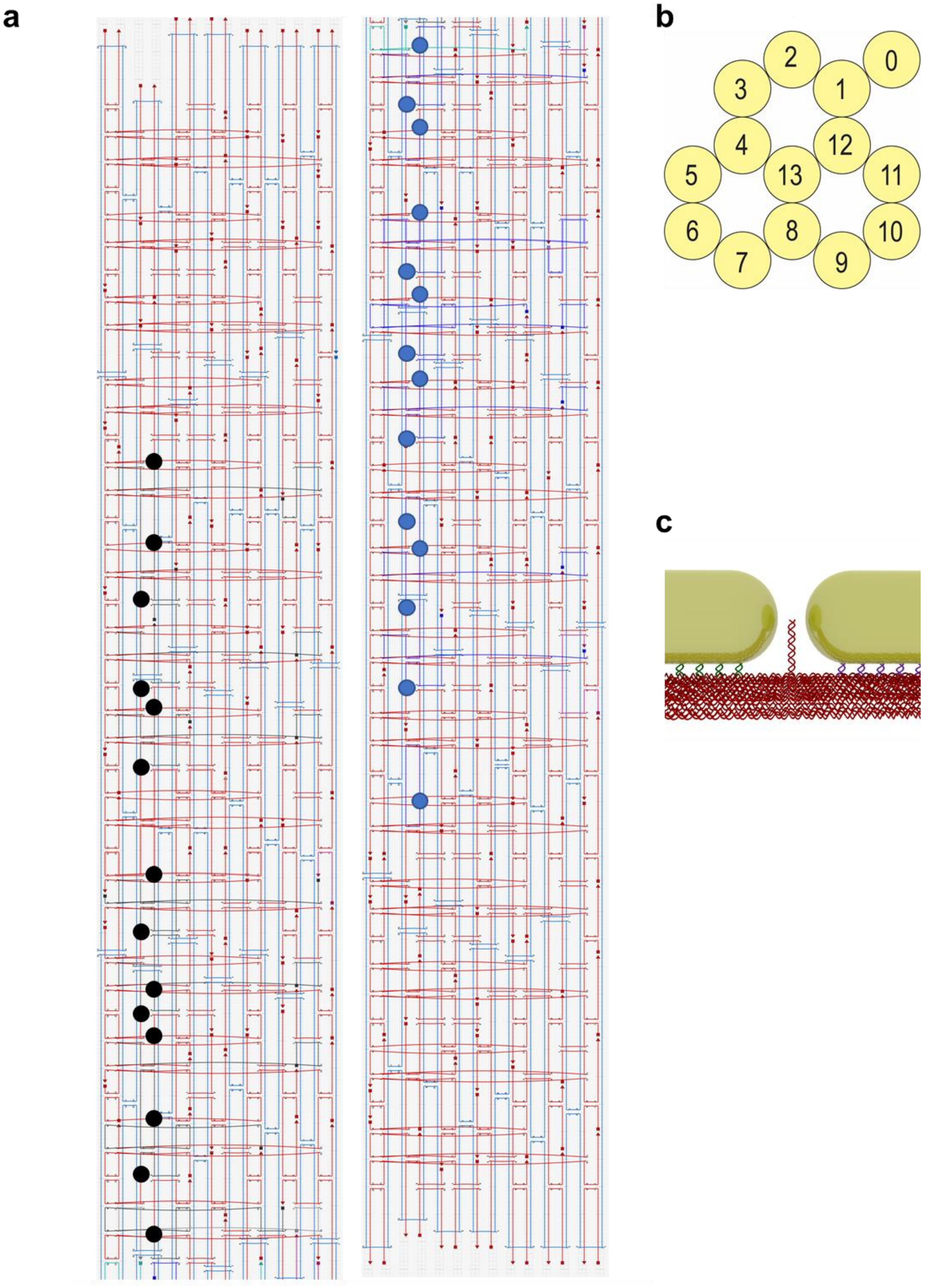

Supplementary Figure 1: DNA origami design. a caDNAno design of 14HB structures. In the schematic picture the scaffold path (blue), the core staples (red) are displayed. Individual binding sides are depicted as blue and black dots. According to the design the structure has dimensions of 215 nm x 12.5 nm and is based on a honeycomb lattice of 14 adjacent ds DNA strands illustrated by **b**. This design ensures a rigid template for the assembly of GNRs (gold nanorods) with enough space for linear conjugation, shown in **c**. To obtain GNR-dimers with tip-to-tip position binding strands were positioned in a long binding sites (60 nm and 14 binding strands per each side). Each binding strand was elongated with a specific sequence, either poly-A (AAAAAAAA) or Random' (ATGTAGGT) that were complementary to the sequences on functionalized GNR-rods (T-19 or Random respectively). To position the molecules of interest in the hotspot, two core strands were elongated at their adjacent 3' and 5' ends. These extended sequences were programmed to be complementary to each other, 20 bp for the dye molecule and 5 bp for the biotin and HD22 aptamer. This design results in a double helix protruding upright from the surface at a defined position, elevating the molecules of interest between the GNRs. We hypothesize that the steric and electrostatic repulsion of the DNA phosphate backbone fosters an upright position with only minor fluctuation around the desired midpoint, as it has previously been observed in super-resolution microscopy experiments [1]. One of the extended DNA strands was labelled with the molecules of interest via a chemical linker. Biotin was used as the in-liquid binding site for streptavidin and potential anti-biotin IgG capturing. HD22 was used for capturing thrombin from solution.

Supplementary Table 1: DNA sequences. The table lists all DNA staples used in the 14HB with their function and length. Core staples are unmodified DNA strands that make up the core region of the 14HB. Random' and Poly-A anchors are staples extended with either a "random" sequence (ATG-TAG-GT) or a poly-A (AAA-AAA-AA) sequence that can bind GNRs functionalized with the complementary sequences. Cy3.5 and dye support staples were used to position a single Cy3.5 molecule in the hotspot. Likewise, HD22 or Biotin staples could be used instead of Cy3.5 staple to position either thrombin or streptavidin and anti-biotin IgG between rods. In that case, the HD22/Biotin support staple was used instead of the dye support staple as a complementary strand to create a double helix.

Staple Type	Staples Sequence	Staple Length
Core staple	GTTGGGATGCCCGTTAGTAAC	21
Core staple	GCTTGTGAAAATGCTTGCGCC	21
Core staple	TGGCTTAAACATGTAGTTTCATTCCACATCAACATTAAT	39
Core staple	CACAACATAATTGTTACGAGCTC	23
Core staple	ATCAGGTAACTAGCGAAAAGCTTAAATTGTAAACGTTAATGACCGGTTG	49
Core staple	AAATTAAATGATATAAGTGCCGAACGCCTTCGCATTAAATTTCAAA	46
Core staple	CTTGAAAGGCTGAACCACCAG	21
Core staple	GTAAATATTGACGGAAATTATGGGCGACA	29
Core staple	TTTAATTTCTCAGAAGCAAAGTAAAACGGATTCATATGCAGA	42
Core staple	TAATTTCCGAATGCTGATGCACAACAATAGATAAC	35
Core staple	CAGGCACAAGAAACTGCCAAGATGAAAGAT	30

Core staple	ACCGCTGCCTTTAAATCAGTTCCCAGTCACAGCA	34
Core staple	AACCGAGAGCCTTTTAATTTTTTCACGTACTTTCATTTCTGT	42
Core staple	AAACATGTTTGCTCACCCTCACATTGACAGATTTTGTTAAAAATCTCAG	49
Core staple	CCCAACCGAGGAAGCTATCACGTTGTTGATTCAGCATTCA	40
Core staple	CAATCCAGTTACAAAATAAACAGCGTAT	28
Core staple	AATCGGCGCGCCAGACAGCTGGCAGCAACCGAAATCGGCAAA	42
Core staple	GGAACCTATTACTTGTACTGGTAATAACTTTTGATAGCGCA	41
Core staple	CCACCCCCCATTAGCAAGGCCACCACCGCACCCTCAGGATTAAATTTAC	49
Core staple	GTACTAGGAGCTAAAAAGATAAAACAGGCATCACCTGGGCGC	42
Core staple	GAAGCTCATGAAAACATATTTTTGAATGGCTTCGCCAACAGAGAT	45
Core staple	TAGTAAATCGCGGAACAAGGGCGAAAAACCGTCTATCAACATTATTCATC	50
Core staple	AGCCCAATCGGATATTCTGACGAGGATGGTTAAAAAAA	38
Core staple	AGCGGGCGCTAAGAATTCGACAGACGGGGAAAGCGCGAAAGG	42
Core staple	AGCCAACGCATTTAGGCAGAGGCCCAATAGTTACCAGTATAA	42
Core staple	CAGATATTACGCCAGCAGTTGGGTAACGCCCGGCCAGAAT	40
Core staple	TAGGTCGAGAATCGTAACATTGCCTGAGAGTCT	33
Core staple	TGAAATTTATATGGAGAGAATGATGACCGTAATGGCGAAAATCCT	45
Core staple	GAATTCGTGCACTTAAGAGTCACGGGCAGGTGGTT	35
Core staple	TCATGGAAATCGAACAAGATGCTGCAAGATTCGCACCAATAAAATTGAG	49
Core staple	ATATGTAGGGCGCATTTTCGAGCGGTAAAGTGTTC	35
Core staple	TGTTCCAGTTTGCCCGAGAGGTGGTTGCGGTCC	33
Core staple	GCGAGAGCCAGACGGGCTCATACGTTAACGGATTGTTCAAATATCGCGT	49
Core staple	AAAATGAAAACAGACCCTGATTCATCGATCAG	32
Core staple	AGTGATCATTGCCAACCTACTCATCTTTG	29
Core staple	ACTATTATAGACCGGGTAAAAAACAGTTCAGAATTTACCCTG	42
Core staple	ATGAGTACATAAACATTGCTGTATCTTAACTTTAT	35
Core staple	CCAGTTTGTTTGCCATCTTGGTTTTCTG	28
Core staple	ATAGGTATAAATGTTCCTGTAGCCAGCTAATAACCATAGCCCGTAGAGA	49
Core staple	CTGAATCCTTAAATTATAGATTTTCAGGTTTAACGTGCCCCCTAC	45
Core staple	AAGGCTCTCGAGGCTTTGAGGACTAAAGAAGAGGCTAATTTCAATC	46
Core staple	CACAGACCACTGAGTCATTTTCAGGGATGTCCACC	35
Core staple	CAGAAAGGCCGGACAGCACATGAGGAAGTTTCGAAGGCATGAATTACGGA	50
Core staple	CTAAGAACGCGAGGCGTCAAGCAATAGGAATCGAAGA	37
Core staple	GCGAACTGAGTGAAACAATAACGGATTCGCCGCAGAGCCCT	41
Core staple	TAGAAGTTCCGGCTACAAAGACTGTTTCTGAATTTCGCGTCG	42
Core staple	TATTCAATTAATTTAATGGAAACAGTGCTT	30
Core staple	AACTTGAGGACTCAATCAATATCTAGAGCCACACCGCC	38
Core staple	GCAGCGAAACTTTTGCCAGCTCATTC	26
Core staple	ACGCTGGTTTGCCCCAGCAACATTGCAAGTTATCAATAGGTCTCTG	46
Core staple	TTTCGTAATGACTGGATAGCGTCCTAATAGTCAAAATA	38
Core staple	TTTTTAGACAGTCTATAAGTTGCTCATTTTTTAACTGGCCTGAGCGAGTA	50
Core staple	GTTAATGCTGAGACTCCTCAGCATTTTCGCCTCAGGAAGATC	42
Core staple	CGTTCTAAATCCTCTGGCCTTGATATTCCAAGAACCACCACC	42
Core staple	CGGGAAAGAAACCAAGGATATCTCAACG	28
Core staple	ACAAATAAGTTTATGCAAGAGAAGGATTAGAGCCG	35
Core staple	TGTCGCGCGAAACAAAGTCTAAAACAAAACGAACTTTTTTCGGAAC	46
Core staple	CTGAACTCTACCTTCAAAAAGTCACGAAGGGCCATATTTAACTATTAGA	49

Core staple	AGCTAATGCAGAACTAAAGTACCGACAAAACAGTAATGGTT	41
Core staple	GTGAGAATAGTGCTATAAAGTGTAAATGCCCGCATTAATG	40
Core staple	TTCCACATAATTGAAACAAGCACGGAAACCAGTTTGCAACGATAAATCA	49
Core staple	GCACTCCCGCGTTTAACCATCCAGTAGCACCATTAGACT	39
Core staple	TACCTTTGCATAACTTGATACTTTCGAGGTGAATTCAAAAGGTAAA	46
Core staple	AACCGCCAGTACCATCATATGTGATACAGGAGGCT	35
Core staple	AGCGTGAGTATTACTGCTAAACTGGAAACTTGTTGCACTGCA	42
Core staple	GAGGGTACTTGCAGCTTGCCCATTACCCTAATCTTGA	37
Core staple	ACGTCAAAAGAAACTGATTATAATAATGGAAGGGTT	36
Core staple	CATATCAGAAAACAAAGTTACTTACCTGAGCAAAAGAAGAAAATGAGTG	49
Core staple	CAAAGGAAATAATACCTTATCATTCCAC	28
Core staple	AGGCGGTCAAAAAATAGCAACAGATTATTTAACGTTAACCATC	43
Core staple	GTCTGAGTTTAACCCTAATGAACGATACTTCGTGTGATAAATTAATTAC	49
Core staple	ACCCAAATCAAGTTTAAATCCTTT	24
Core staple	AAACAGAAATAGAAGAAACATCATAAAACGAAGGGTTTAGCGA	43
Core staple	GAGCCGAGTCAGAATAGGAACAAGAGTCCACTATTAAAGATTATA	45
Core staple	CAGTTGAATCCTGATTGTTTGGATTATTAAAACATAATTTTGCTGC	46
Core staple	GTAGAAAATAGTAACGGAATA	21
Core staple	CTCCAAAGAATACAACAACGGAGATTTGCTAAACATGAAAAT	42
Core staple	ATCAATCACTCATCGGACCTGGAAGAGCGCATCGCTAAACCA	42
Core staple	CTAATCTAAAATAGCCATTCCGAACGAGAATACG	34
Core staple	CATAATTGTTTTCAAAATCATGTTGTGCAGAACCA	35
Core staple	TATTTTTGGGGTCGAGGTGCCGTAAGATTTAG	32
Core staple	AACAAATTTCAAAAAATCGCTGATTGCAGTAAC	33
Core staple	AATATAATACATTTGATAAAAGTATCGTCTAGGGAC	36
Core staple	AAATATAATTTGTTTATCCTCCCG	24
Core staple	CGCCGACAATAAAACGAGTAGTTATCAGCTTGCCGATAGTTG	42
Core staple	TGGCACTTCTGACCGACCAGTAATAAAGAAATGGGAAA	38
Core staple	AAATACCGACGCGAGAAAACTTTTTCTCGCAAGTAGGTTGAAGAGAAAACGCCA	54
Core staple	CGTAACAAAGCTTTCTGGCTGACCTTCAACAGACCAGGCGCATTG	45
Core staple	ATGCTTTTGCGGGAGAAGATTATGAAAGCTAAAAGGCAATTCTACT	46
Core staple	GCCCGACATTGGCAGATTGTACCGCCATCTTTACTGAA	38
Core staple	ATTAATCGGGGAGAGCAGATGAATATACTTTGAATAACTCAC	42
Core staple	GCAAGCTGGTCGCGATCCGCTGCCACGCAACACAGC	36
Core staple	ACGAGGCGCATAAAAGGAATTACGAAATGTTACTTAGCCCT	41
Core staple	GGAATTGGTGAGAATCCAGACGTTAGTACTAAAGTCCCTCAG	42
Core staple	GGGGAGAGAACTAATTTGCTGGCATTCGCAT	31
Core staple	GTCACACTGAAAGCGCGAGTAAAAGAGATACTTCTTTCACCA	42
Core staple	TTGTCTCCATGGTATAGATATACTGTCGTGCTCTCCGGTGTC	42
Core staple	AATAGGGATTACTTTATTGCGATTATGGCGAAAGGGGGAAGGG	43
Core staple	GTCTCTGGCGGGGTAAAGTATTATTAGCGAGGGGACGACGAC	42
Core staple	AGAACCCAGACAATCGCCATTGTATTAAGCAGCAAAT	37
Core staple	ACATGTATCAACAGGTATCATATGCGTTAAACACCTAACT	40
Core staple	TATCCCAAAAACAGGAAAACGGTAGGGTTGAAAATCACTAATGGG	45
Core staple	CAGGGCTGAGGGCAACGGCTACATTAAACAGCCGATATAAGAACGAAGCA	50
Core staple	ATACAGATGAACGGTGTTCAAGAGAAATCAA	31
Core staple	AGACTTTACAAACCACTAACAA	22

Core staple	AGCCTCATCAGGAGGTAGGTAATGCAACGTCATTTTTGCGGA	42
Core staple	GTACCGTAAAGCCCTCATAGTCAACTTTGAACAGGAACCCAT	42
Core staple	AGTACCTATTGCGTTTGCACGACTTCTGCAGATGATGGCAATCATT	46
Core staple	CTCAGAACGCCCACAATTGCTCCTTTTGATAAGAGTAGACAA	42
Core staple	CGCATCGCGGCGGACCGTCGGAAAATAATTCGCGTCCAA	39
Core staple	ATCGATGGATCCAATAAATCATACAGGCATCGGTTGGAATAGTGAG	46
Core staple	CCGGAAACCAGGCAGGGCCTCTTCGCATTAACTCT	35
Core staple	GTCCAGACATTACCAGAATTATACCAACGCTAAATATACAA	41
Core staple	AGCTACCTAGCGATGAGTGAAAGGAGCGGAACGTGGACTCCA	42
Core staple	TACATAACGCCAGGATTACAGGTAGAAAAACTAACCGAT	39
Core staple	GAAAAGTGTGGTTCAGGGTTCATACATAA	29
Core staple	TTGACTAGCGTAACGATAATGAATACAGTTT	31
Core staple	AGAGCCGAGACGATATTAAAGCCAGAATGGAAACCGGTTGAG	42
Core staple	GCTATATATAGATGTAAGAGCTGCGTGATCGGTGCAAGCGCCATTCGCC	49
Core staple	AGTATCGGGTCATAAGCCGGAAACGTCA	28
Core staple	GTAGATGAAGCAGACAATTGGGCGGAGTTTTTAGCTG	37
Core staple	AAAGTAGCTCGAGCTTAATTGCTGAATCACGTTGGTGTAGATGGG	45
Core staple	GATCGGTTTTGTAAAAGATAAGAACAACATAATTCT	36
Core staple	ATTCAGGCTGCGTCAGGATGCAGGTGAGTTGGAGGATCATTATTTCTGC	49
Core staple	AAAACGACCATTTTCTGCGGTACGGTGTCTGGATTTAAATAAGATTCGCA	50
Core staple	GAAAATCAAACCAGGTTATAGATTAGGAAGTATT	34
Core staple	GTCTGACACGAGCATGTAGATAGACCTT	28
Core staple	CTTTATAAAGAACTACGATAAAGGCATAGT	30
Core staple	GGCAGTTACAGCTCATTGTTGCCAAAGACA	30
Core staple	GGCCCACTACGTGATTAATTTGGATTTAAGCCGTCAATAGATAAAG	46
Core staple	ATCAGTTTAGCTATGTAGCATTAACATTGTATAAGTTGTTAA	42
Core staple	CCTGCTCCACAAAATGTTTCGATTATACCAAGAAATCCGCGA	42
Core staple	CAAGAAAGAGGAAGGGAACTTTGTATCATCGCCTG	35
Core staple	CAGCGGACGAATAAAATTGTATCGGTATACCGCCATTTGTCGAGCATTC	49
Core staple	ACCCACAAAATTTGTGCGAACGAGTCGGCATACAAATATTTCCCCATTGA	50
Core staple	ATGGGATCGAACTGGGCTTGAAAACACCTTCGGTCTCAGGATTAGAGAG	49
Core staple	ATAATCAATGTCAAGGCGGATTCAACCGGAACAAATAACCGTGCATCTG	49
Core staple	TTTCTTTCAGCTGCTTTCCAGTCGGGAAGAAA	32
Core staple	ACAACCGTACGAGCATACCCTGTAATTACAACGCCTGTTCAATAA	45
Core staple	TGCGCCGCTACATGGGGGGCGCTGGCAAATCCAGAGGAGGCCTTATAAT	49
Core staple	TTACGAGAAACGCAATCAACGATTGAGGGAGAA	33
Core staple	ACCCCCAGAGCCACTACCATTAAACCTCA	29
Core staple	GCAGGTCCCGCCAGGAGCCACGAACCGCATCACCGGA	37
Core staple	GCAAAATTAAGCTTTAGAAAGGAAGGTGGCATCAAAGAATTA	42
Core staple	GAGACGCTACGATCGGGTAGCGATGCCAGAGTCTGTAGTGTCAGGTGT	48
Core staple	TATGGGAACAAGGGATCGCGAGCTTCAAAGCGAAC	35
Core staple	ACTTGCGGGAGGTTTTCACCCAGCAGA	27
Core staple	CAACCATCCGCCACCCTCAGTGTTTAGTTTAGTTTAACTAAA	42
Core staple	TTAAGACAACAAAGTTTAAGAGCCATTTTGAATTATCACCGTACGGAAG	49
Core staple	AATAGTAATTTTCAAATGGTCTTTATAACAGTTGATTCCCAAAGAT	46
Core staple	TTTGGAAGCCTAGTCCGTGATCCGGTATT	29
Core staple	TTCAACTAGAAAAAGCCCTTTTACCAGACTGTAGAGCCAGCTTTCCGGC	49

Core staple	ATAAAGACGGTCAACTTTAAATAAGGGGAGTTACCGGAAGCAAACTCCAA	50
Core staple	ATCCCATGCCCTGAGAGAGTTATTGCCCATTAAGAGTGCCTATTCCA	47
Core staple	AGCCGCCACCCAATTCTCCGTTTCATAATCAAACTCCCTCAG	42
Core staple	TGGCAAATAAAGAATTTACATCGGGAGCCACGCTGGGTCAGT	42
Core staple	GTATTGGCAACGCGTGCGTTGCGCTCACGCCTGGGCGCT	39
Core staple	TTAAAGGGGGAATTAGAGCCGTTACCGATTCATA	34
Core staple	CCTCAAATAATCTAAAAGGTG	21
Core staple	ACAGTTCAAAAATGCGCCGCATGGGAGTAAACGTATTAACCA	42
Core staple	ATTGTGCTGGGGATGAGATAATATCAAGTTCGCCATCCTGGGAGCG	46
Core staple	CAGTGAGGCCACTAAACCACCAGCAGCAACAATATATTAGTATCG	45
Core staple	TTGTCGCTATACCTTTTTACATTTAACAATTATTAGTCTTTAAGAAT	47
Core staple	TAATTGAGAATCTGGGTTTATAAAAAGCCTGTTTATAGGGCT	42
Core staple	TATGAAGGTGTTATCTCCAGCAAAGAATTGACAATGCAGTCAACT	45
Core staple	TTTAAATAAAAGGGGTGTATCTTTCGCATTTGGGGCGCGAGCTG	44
Core staple	ATTCTGATTTGAATTTATTTTGACGCTCATTGAGTAGGGCGAT	43
Core staple	ATATAGAAGGCTTAAGAAGCCGTAGTACCGAATCGGC	37
Core staple	TGCAACATAGCCCTGAAATACCTACAATGAAATCAA	36
Core staple	TGGTTTACCAGCGTGTAGCAATAACGCAACAAGTTTTCTGGTG	43
Core staple	ACGGGTATTAAACCATTTTATTACAAAGTCTA	32
Core staple	AGCAGATTATCAGATTGACGCTACGGGGTATCTGCATATGAT	42
Core staple	CGGGGATTTATGCCCTCTCTGTACAAAGGTGGAAAGACAAACCATGAATTTAT	53
Core staple	TCTTCACTGAGTATATACCGTATAAATGAGTATCA	35
Core staple	AATCAAAACAGGAGAATGGGTAGCTTAGTTCACCGGGTTTGC	42
Core staple	ATTCTTTAGCGAACCTGAATCTACTGAACGGAAGCGCAAGAAAGAGTGA	49
End cap	CCCCAAAGGAAGGGAAGAAACGGCGAACGTGGCGAGCCCC	40
End cap	CCCCGTGCTTTCCTCGTTAGAATGGGATTTTAGACAGGAACGCCCC	46
End cap	CCCCAAACTATCGGCCTTGCCTGAGTAGAAGAACTCCCCC	40
End cap	CCCCATTCATTGAATCCCCCCGGAATCGTCATAAATCCCC	40
End cap	CCCCCCTAAAGGGAGCCCCCAGCACTAAATCGGAACCCCC	40
End cap	CCCCTTGGGAAGAAAAATCTTATACCAGTCAGGACGCCCC	40
End cap	CCCCACCACCACACCCGCCGGGTCACGCTGCGCGTACCCC	40
End cap	CCCCGTACGCCAGAATCCTGAGAAGTGTTTGATTAAACAGAGCGTGGTT	49
End cap	CCCCATACCACATCAACACTATCATCCCC	29
End cap	CCCCAACCCTCGTTTAGCTTTTGCAAAACCCC	32
End cap	CCCCAATCAAAAATCAGGTCAACGAGAATGACCATACCCC	40
End cap	AAGAGTCAACTACAGTTGAGATTTAGGACCCC	32
End cap	GCTTTGACGAGCACGTATAACCCCC	25
End cap	CCCCGAAGTTTTGCCAGAGGGGGAATACTGTCAAATG	37
End cap	CCCCATTAACCGTCACTTGCTGGTAATGTGTAGCCGCTTAA	41
End cap	CCCCAGGAAGCCCGAAAGACCATCAAAAAGATTAAGCCCC	40
End cap	AGCTTACATAACATTGTAGCATCTGTCCATCACGCAACCCC	41
Random' anchor	CCAGTACAAACACCTGAGTAATGAGCCACCACCCTTTCGTCAATGTAGGT	50
Random' anchor	TGCCTAACAGTAAGCGTCATACATGGGTTTTAACCTATTTCATGTAGGT	49
Random' anchor	CCAATGATCATCGGATAGCCGTCCCCGTATAAACAATGTAGGT	43
Random' anchor	ACAACTTGACCGCATCAATTGCCGGAGAGGGAAAATGTAGGT	42

Random' anchor	TGAAAAGTAATTTGTCACAAAGACACCACGGTGAGTAACAGTGCTTAATGTAGGT	55
Random' anchor	GAGATCTACAAAGAGCTGATATGTAGGT	28
Random' anchor	GGAGCAAACAACCAAAAACCCTTTATCATATATATGTAGGT	41
Random' anchor	CGAAGCGGCAAGAGAGGGAGGATCTGATGTAGGT	34
Random anchor	GCTGGCCGGAGAACCCTTTCAACGCAAGGATAATAGCTATTTTTGAATGTAGGT	54
Random' anchor	AGGTGGCAACATATAAACAGTATGGGCATGAATGTAGGT	39
Random' anchor	GAACTGGAAACTGCGTGCGAAGACGCTTGCCAGTTCGAAGATGTAGGT	48
Random' anchor	TGAGCGAAATTAATCGGACTTGTATGTAGGT	31
Random' anchor	ACCAGGCCCCCTTAAGAGGCCCCCTGCGGGGTCAGATGTAGGT	43
Random' anchor	AAATCACGATAGCAGCACCGTGCGTCAGAAGGAAAAAGAACTTTAGCAAACATGT AGGT	59
Poly-A anchor	GACAATATCCTGAACATTAGAATTTGTTTAACGTCAAAAAAAAA	44
Poly-A anchor	CCATATTATTTACAGCCTTTATGAATTGGTAACATCAAACGCAAAAAAAA	50
Poly-A anchor	GCTGGCCGGAGAACCCTTTCAACGCAAGGATAATAGCTATTTTTGAAAAAAAAA	54
Poly-A anchor	AGAATGTATCATCATATTCCCACCAGATAACCTTACATAAACATCAAGAAAAAAA AAA	58
Poly-A anchor	CAAATTATCACGAGTACACATATACGCACTCGAAAAAAAA	40
Poly-A anchor	TGTTTATAATCCAAAAATATAAATGGTTTGAAAAAAAA	38
Poly-A anchor	TGCCTAACAGTAAGCGTCATACATGGGTTTTAACCTATTTCAAAAAAAA	49
Poly-A anchor	TGAGCGAAATTAATCGGACTTGTAAAAAAAA	31
Poly-A anchor	TGAAAAGTAATTTGTCACAAAGACACCACGGTGAGTAACAGTGCTTAAAAAAAAA	55
Poly-A anchor	GTGAACGAGCCGGAAGCGATACCGTGATTAGCTGTTTCCTGTAAAAAAAA	50
Poly-A anchor	TGTCTTTTCCCATCTAACATAAAAATAGTCCCAATCCAAAAAAAA	45
Poly-A anchor	TGATGAAACAAATCAATATATATCCCCGGGTACTCCGCTCACAAAAAAAA	50
Poly-A anchor	GAGATCTACAAAGAGCTGATAAAAAAAA	28
Poly-A anchor	AAATCACGATAGCAGCACCGTGCGTCAGAAGGAAAAAGAACTTTAGCAAACAAA AAAAA	59
Cy3.5 staple	[Cy3.5]ATGCATGCATGCATGCATGCAGTAAACGAGCAGGGTTAAGCTTCACCC	48
Dye support staple	TATCGACTCAATGGTGACGACTGGAACTGCTGGTCGCATGCATGCATGCATGCAT	55
HD22 staple	AGTCCGTGGTAGGGCAGGTTGGGGTGACTACTGAAGTAAACGAGCAGGGTTAAGC TTCACCC	62
Biotin staple	[Biotin]ACTGAAGTAAACGAGCAGGGTTAAGCTTCACCC	33
HD22/ Biotin support staple	TATCGACTCAATGGTGACGACTGGAACTGCTGGTCTCAG	39

Supplementary Figure 2:

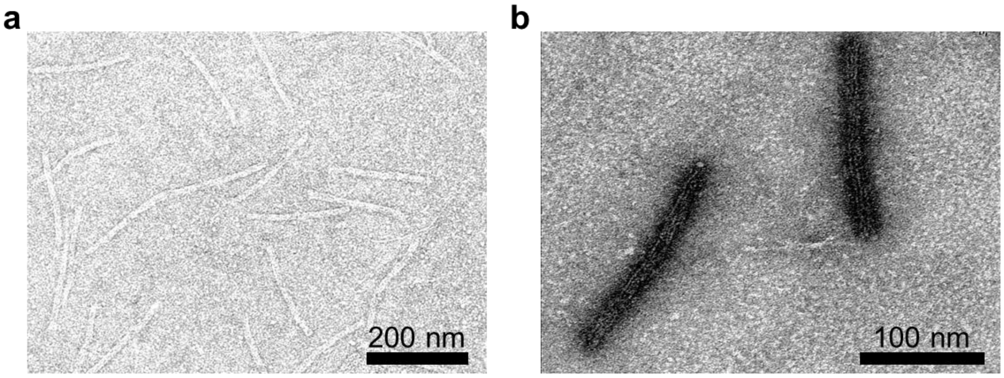

Supplementary Figure 2: DNA origami used for gold nanorod dimer synthesis. a Overview TEM image of DNA origami beams. **b** Close up TEM image of individual DNA origami beams.

Supplementary Figure 3:

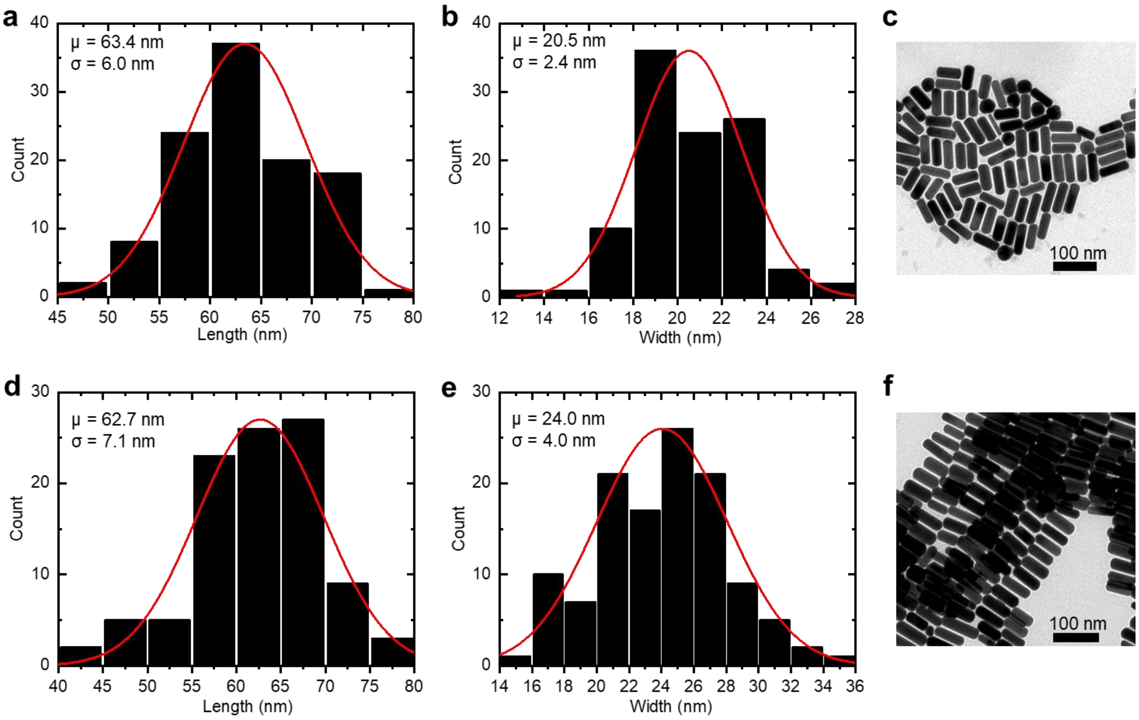

Supplementary Figure 3: TEM of gold nanorods. a, b Length (n = 105), and width (n = 104) distributions of gold nanorods from batch R1. **c** TEM micrograph of batch R1 - gold nanorods. **d, e** Length (n = 101) and width (n = 120) distribution of batch R2 gold nanorods. **f** TEM micrograph of batch R2 - gold nanorods. The red curves represent gaussian fits, with the corresponding mean (μ). and the standard deviation (σ). No nanorods were excluded from the statistical evaluation.

Supplementary Figure 4:

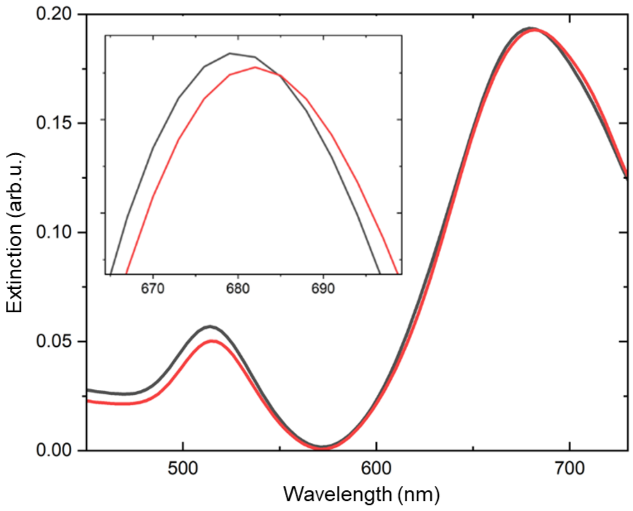

Supplementary Figure 4: Effect of DNA coating on the GNR plasmon resonance. Bulk extinction spectra of gold nanorods before (black) and after DNA functionalization (red curve). Inset: zoom in on the position of longitudinal plasmon peak. The plasmon peak shifts from 680 to 682 nm due to the deposition of DNA on the nanorod surface.

Supplementary Figure 5:

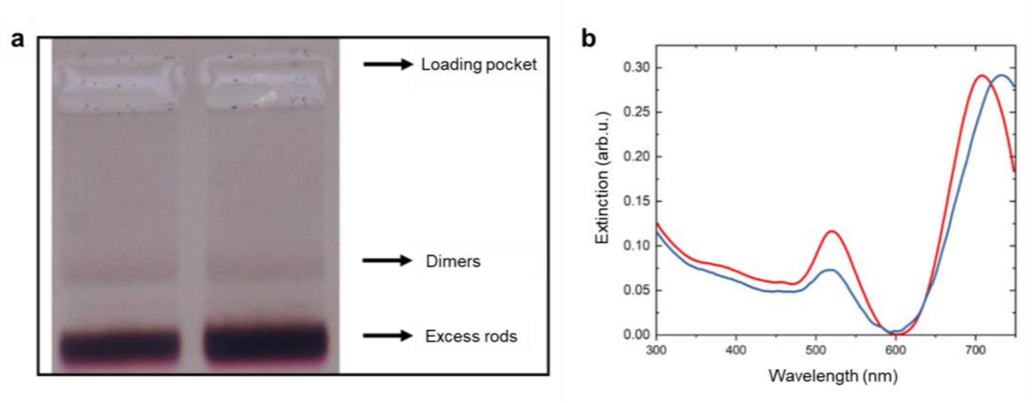

Supplementary Figure 5: Dimer purification. a Agarose gel imaged with white light. The loading pocket with aggregates is situated at the top of the image. Unbound gold nanorods appear as dark red bands at the bottom. An additional band appears resulting from the binding of gold nanorods (GNRs) to DNA origami. This band containing the target structures is extracted from the gel for further experiments. **b** UV-Vis spectra of single GNRs (red) and corresponding dimers (blue).

Supplementary Figure 6:

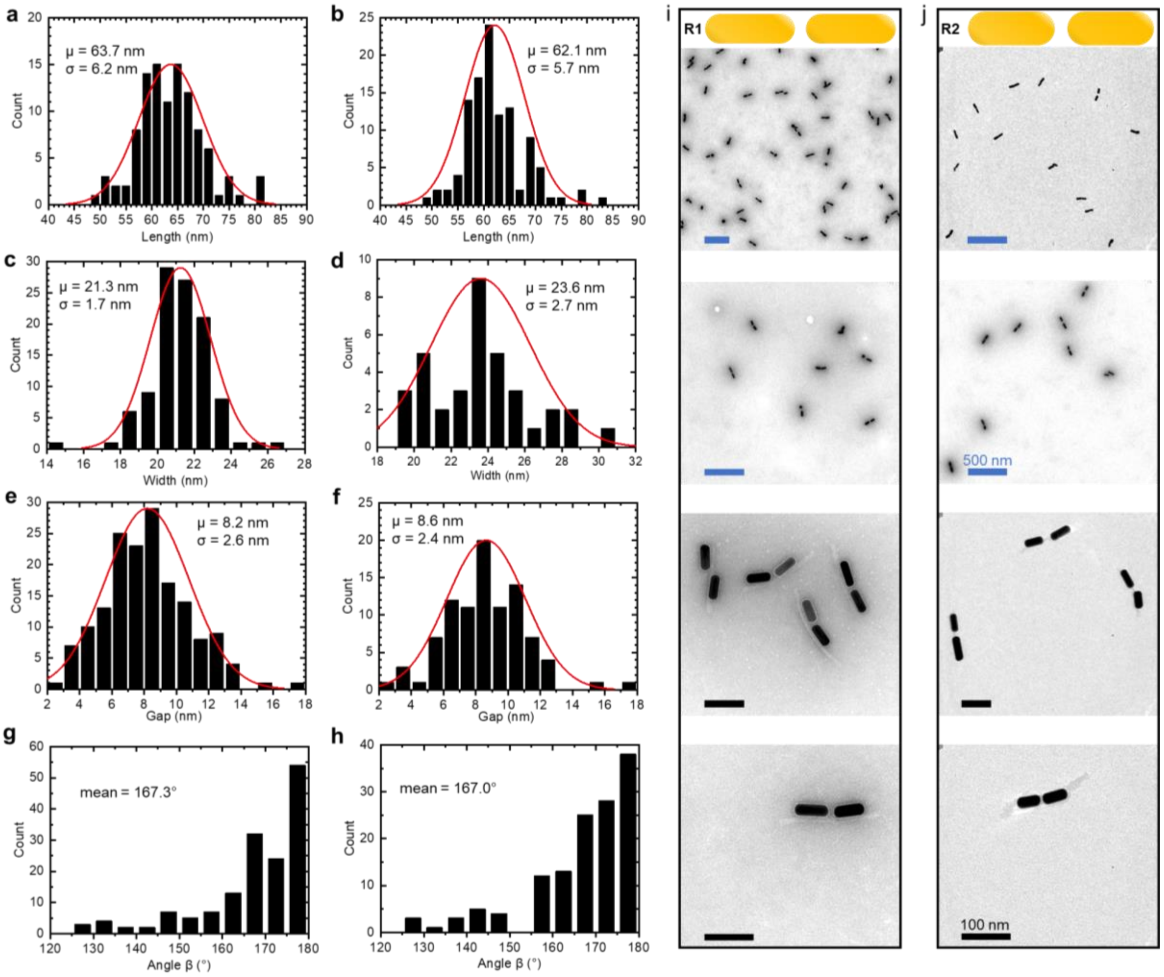

Supplementary Figure 6: TEM characterization of DNA assembled gold nanorod dimers. a Length distribution of gold nanorods assembled into dimers on the DNA origami (batch R1, n = 105). **b** Length distribution of gold nanorods assembled into dimers on the DNA origami (batch R2, n = 110). **c** Width distribution of dimer forming gold nanorods (batch R1, n = 105). **d** Width distribution of dimer forming gold nanorods (batch R2, n = 36). **e** Gap size distribution (batch R1, n = 162). **f** Gap size distribution (batch R2, n = 93). **g** Angle distribution of dimer nanorods (batch R1, n = 153). **h** Corresponding angle distribution for R2-nanorod dimers (n = 133). In the analysis, 180° corresponds to a perfectly aligned nanorod dimer. Only nanorod dimers with an internal angle ≥ 120° between the GNRs were considered here. **i** and **j** TEM images of gold nanorod dimers of batch R1 and R2, respectively. Blue scale bars correspond to 500 nm. Black scale bars correspond to 100 nm. The red curves represent gaussian fits, with a mean (μ) and standard deviation σ.

Supplementary Figure 7:

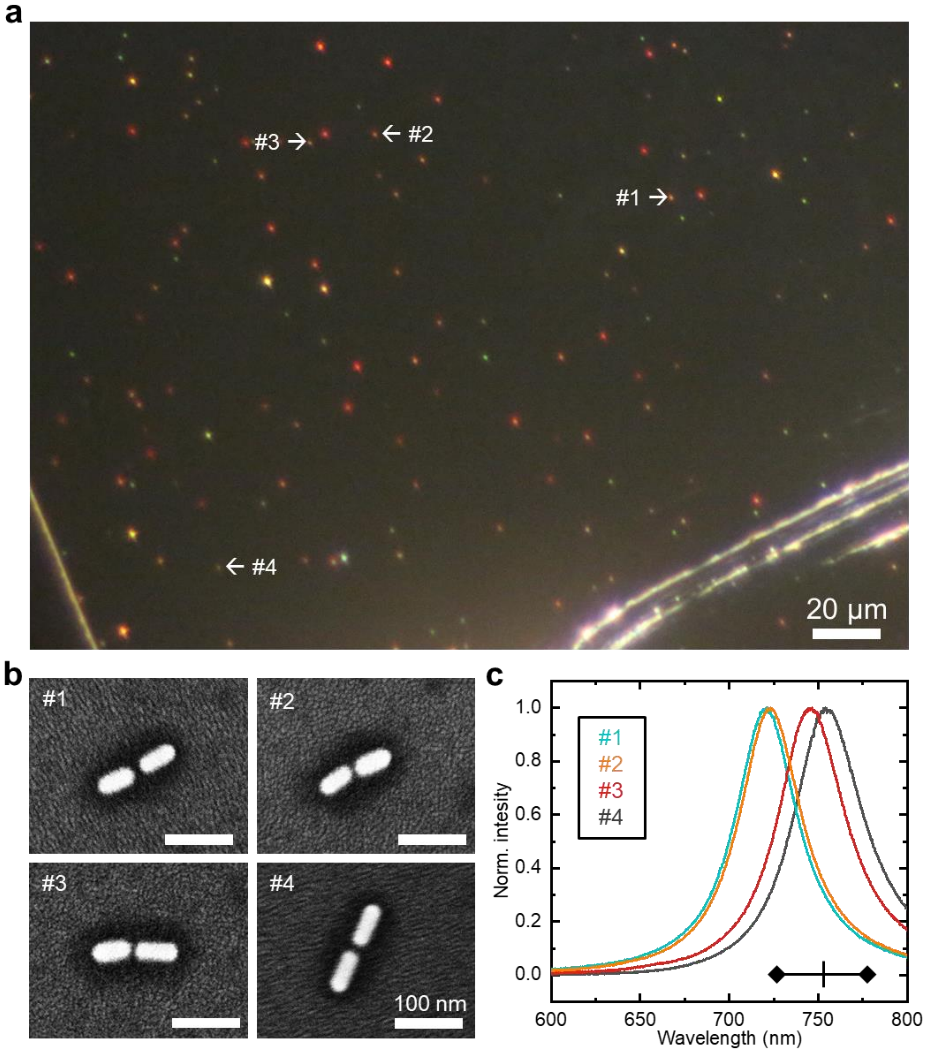

Supplementary Figure 7: Gold nanorod dimer samples characterization. a DFM image of sample surface. Individual nanoantennas are identified by red/orange spots. The nanoantenna density is ≈ 1/400 µm². **b** SEM images of exemplary gold nanorod dimers from a. **c** Dark-field scattering spectra from antennas in a and b, in air. Nanorod dimers from the sample, featured a mean scattering peak at 753±26 nm, indicated by the black marker with error bars at the bottom of the graph.

Supplementary Figure 8:

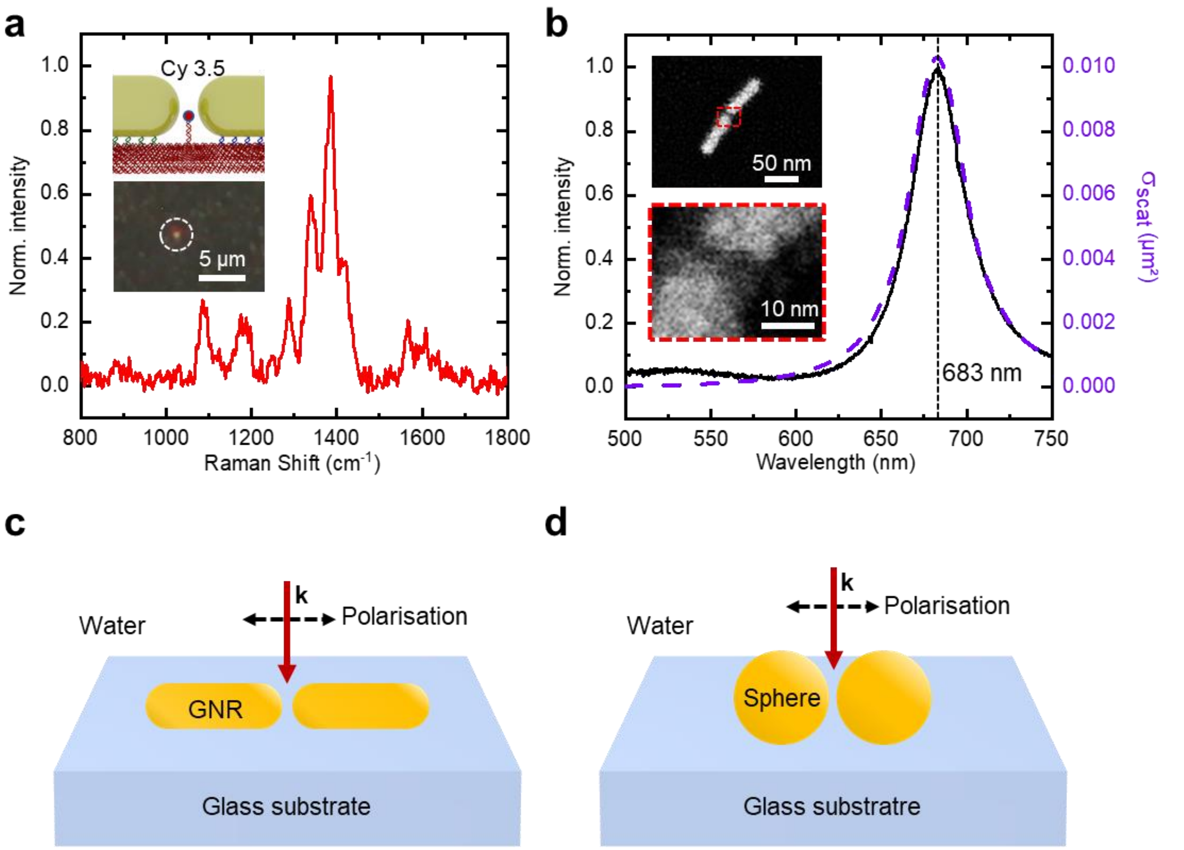

Supplementary Figure 8: Optical and numerical dimer characterization. a Cy3.5 SERS spectrum from a single gold nanorod dimer (DFM inset) in air. **b** Measured (red) and calculated (blue) scattering spectrum of the nanorod dimer used for SERS measurements in a. 64 nm x 21 nm nanorods separated by 7.5 nm were assumed for the calculation to reproduce the longitudinal plasmon mode of the scattering spectrum. Insets: SEM images of the corresponding nanorod dimer indicating a gap size between 6-8 nm. **c** Simulation setup for gold nanorod dimers. **d** Simulation setup for gold nanosphere dimers. For Simulations in a and b, gold particle geometries were approximated with spherical end-caps, and located directly on glass slabs, with an aqueous environment. The red arrow illustrates the plane wave's propagation direction which is perpendicular to the substrate, whilst its polarization is parallel to the long axes of the plasmonic antennas. Source data are provided as a Source Data file.

Supplementary Figure 9:

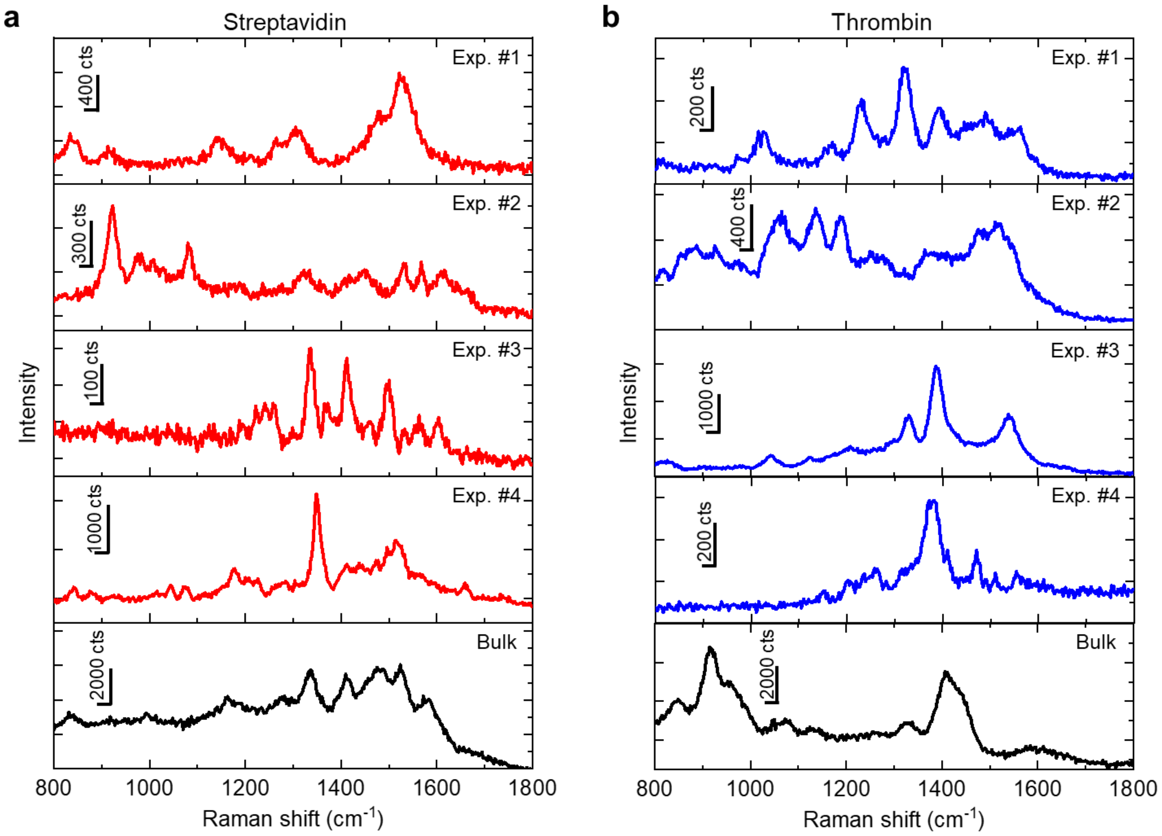

Supplementary Figure 9: SERS spectra of single proteins for several dimer structures. a Additional SERS spectra of streptavidin (raw data) from DNA origami-assembled gold nanorod dimers (red) and bulk Streptavidin (170 µM, dried film) measurement (black). **b** Additional SERS spectra (raw data) of thrombin from DNA origami-assembled gold nanorod dimers (red), and bulk thrombin (610 µM, dried film) measurement (black). Source data are provided as a Source Data file.

Supplementary Figure 10:

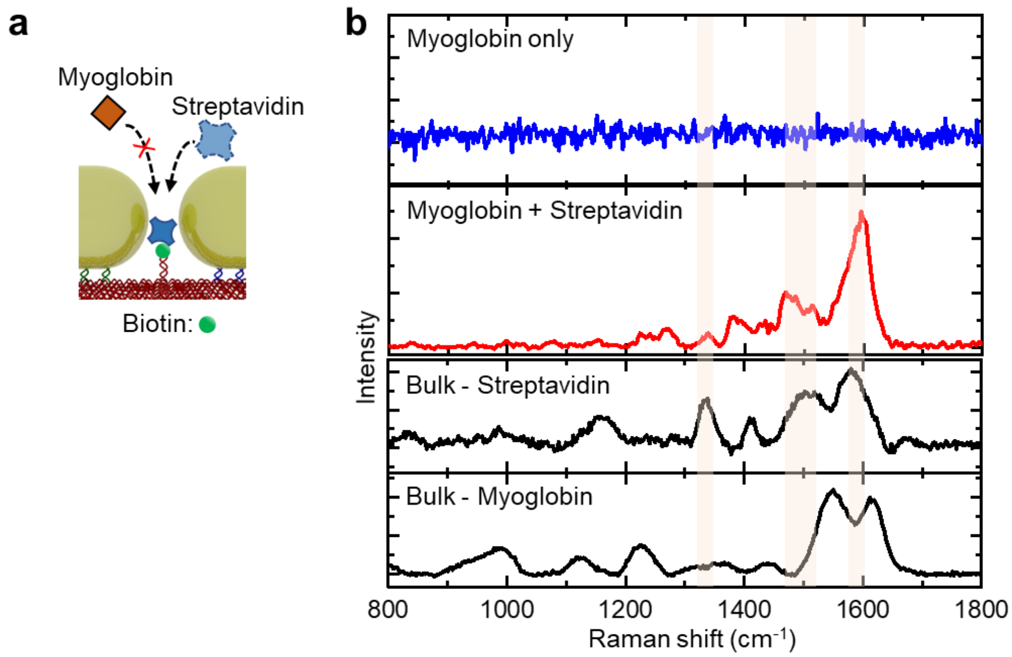

Supplementary Figure 10: Binding specificity measurement. a Sketch illustrating the myoglobin/streptavidin specificity measurement with biotin equipped gold nanorod dimers. **b** SERS (integration time 0.5 s, 1 mW laser power) measurements with only myoglobin (top) and a combination of myoglobin and streptavidin in aqueous solution (second from top). Bulk Raman spectra of streptavidin and myoglobin are shown for comparison. Source data are provided as a Source Data file.

Supplementary Figure 11:

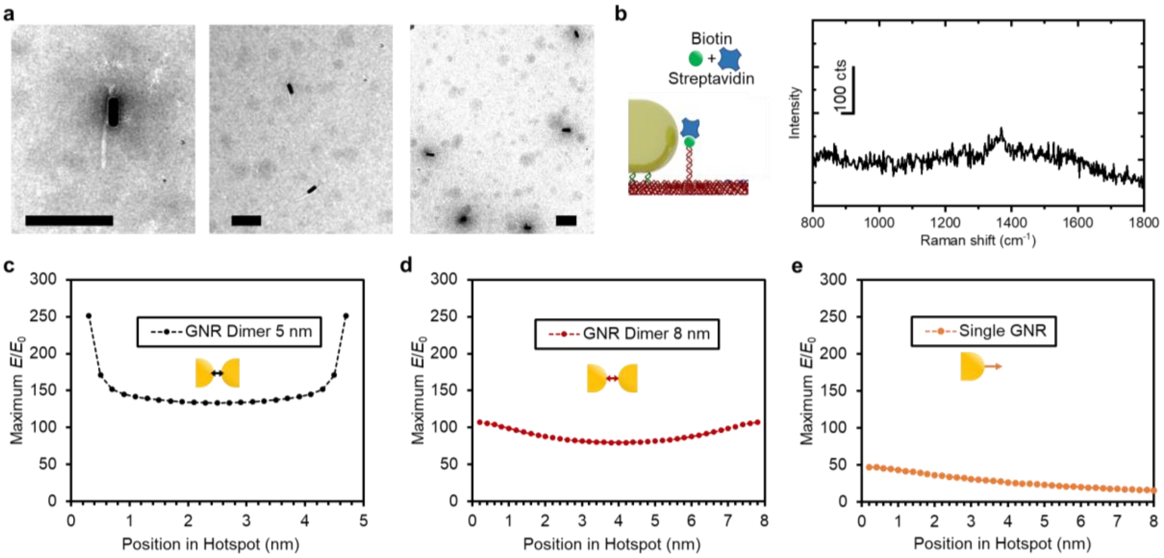

Supplementary Figure 11: Single nanorod SERS and E/E_0 comparison. a TEM images of single gold nanorods (GNRs) attached to the DNA origami beam (scale bars: 200 nm). **b** Example SERS spectrum (in counts, 0.5 s integration time) obtained from a DNA origami nanorod monomer (biotin and streptavidin). Only a weak signal at ≈1370 cm^{-1} is observed, corresponding to ring breathing modes of T, A, G [2]. **c** Calculated maximum *E*-field enhancement across the central axis of a 5 nm gap of a gold nanorod dimer. **d** Maximum *E*-field enhancement across the central axis for an 8 nm gap. **e** Distance dependency of the *E*-field enhancement for the tip of a single gold nanorod. 64 nm x 21 nm gold nanorods were assumed for all calculations. Source data are provided as a Source Data file.

Supplementary Figure 12:

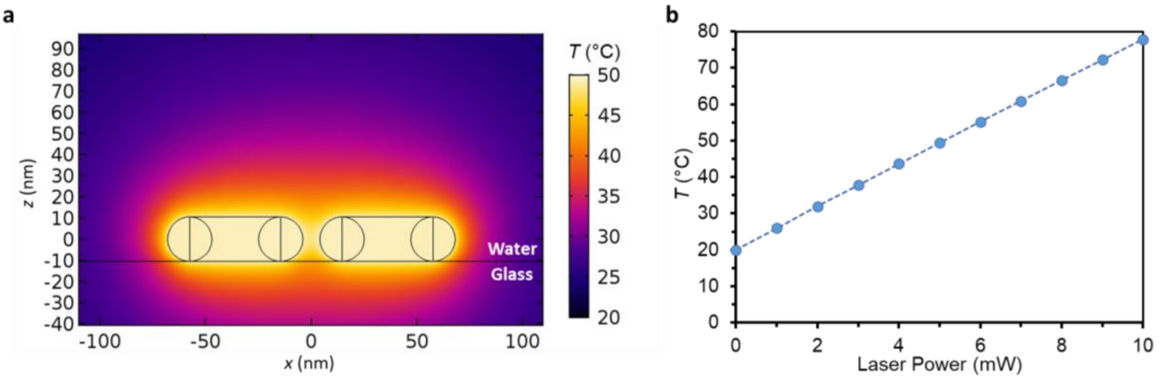

Supplementary Figure 12: Heating Calculations. a Calculated temperature distribution for a 21 nm x 64 nm GNR dimer with an 8 nm gap on a glass substrate in water. Here, focused illumination with a circularly polarized, 5 mW laser (λ = 671 nm) to emulate experimental conditions was implemented. **b** Calculated dimer temperature *T* vs. laser power. Heating powers (derived by FDTD) were applied uniformly in a finite element model (COMSOL Multiphysics) assuming a circular polarized laser beam with a FWHM of 560 nm (measured by camera).

Supplementary Figure 13:

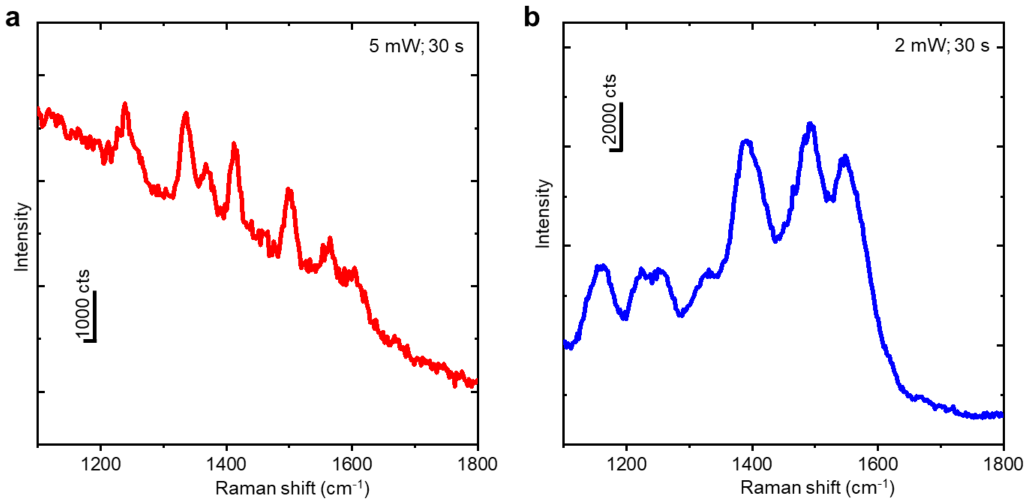

Supplementary Figure 13: Protein SERS spectra from longer integration times. a Streptavidin and **b** thrombin SERS spectra, obtained with 30 s integration times show distinct, assignable peaks (excitation wavelength: 671 nm, intensity in counts: cts). Source data are provided as a Source Data file.

Supplementary Figure 14:

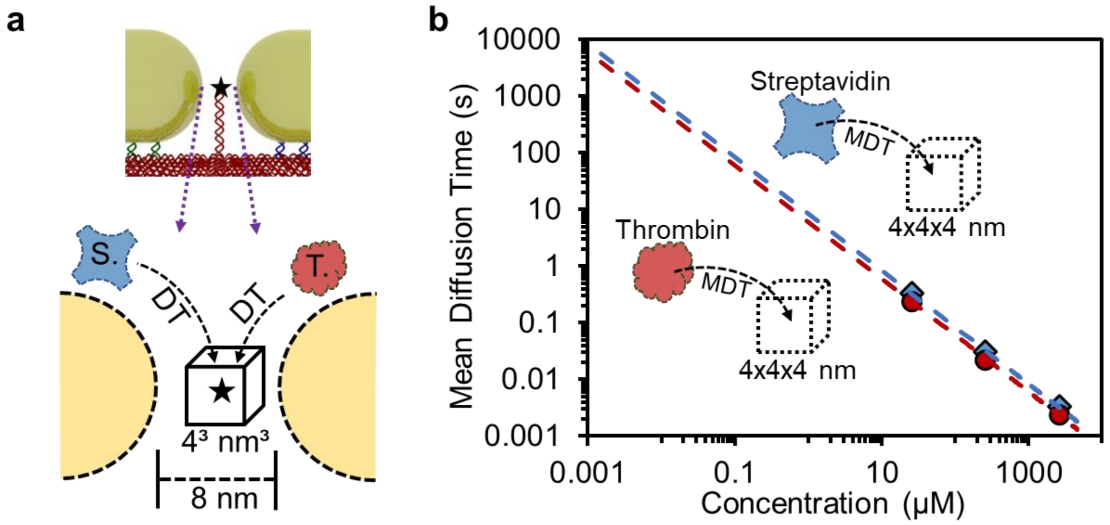

Supplementary Figure 14: Numerical approximation of diffusion times. a Illustration of the mean diffusion time (MDT) modelling assumption of 8 nm wide gaps with a binding site (star) at the nanogap center. **b** Calculations of the concentration dependent mean diffusion times for streptavidin (blue) and thrombin (red) entering the hitbox (100 simulations for each data point). The inverse relationship between concentration and MDT was extrapolated to cover experimental and physiological concentrations.

Supplementary Figure 15:

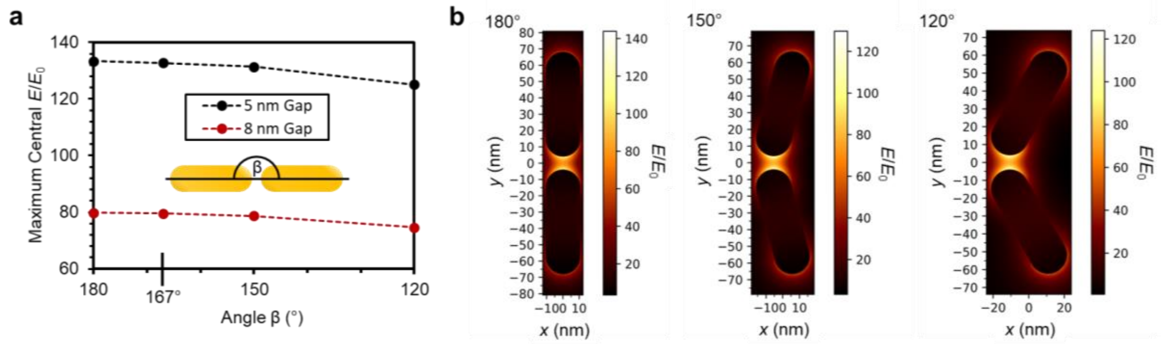

Supplementary Figure 15: E/E_0 dependence of the GNR alignment angle. a Calculations of the maximum central E/E_0 for gold nanorod dimers with 5 nm and 8 nm gaps. An average alignment angle of 167° (indicated in the graph) was found for synthesized dimers (see Fig. S6 G, H). **b** Map of the peak E/E_0 value for gold nanorod dimers (8 nm gaps) with alignment angles of 180°, 150°, and 120° (excited at 792 nm, 792 nm, and 790 nm respectively).

Supplementary Figure 16:

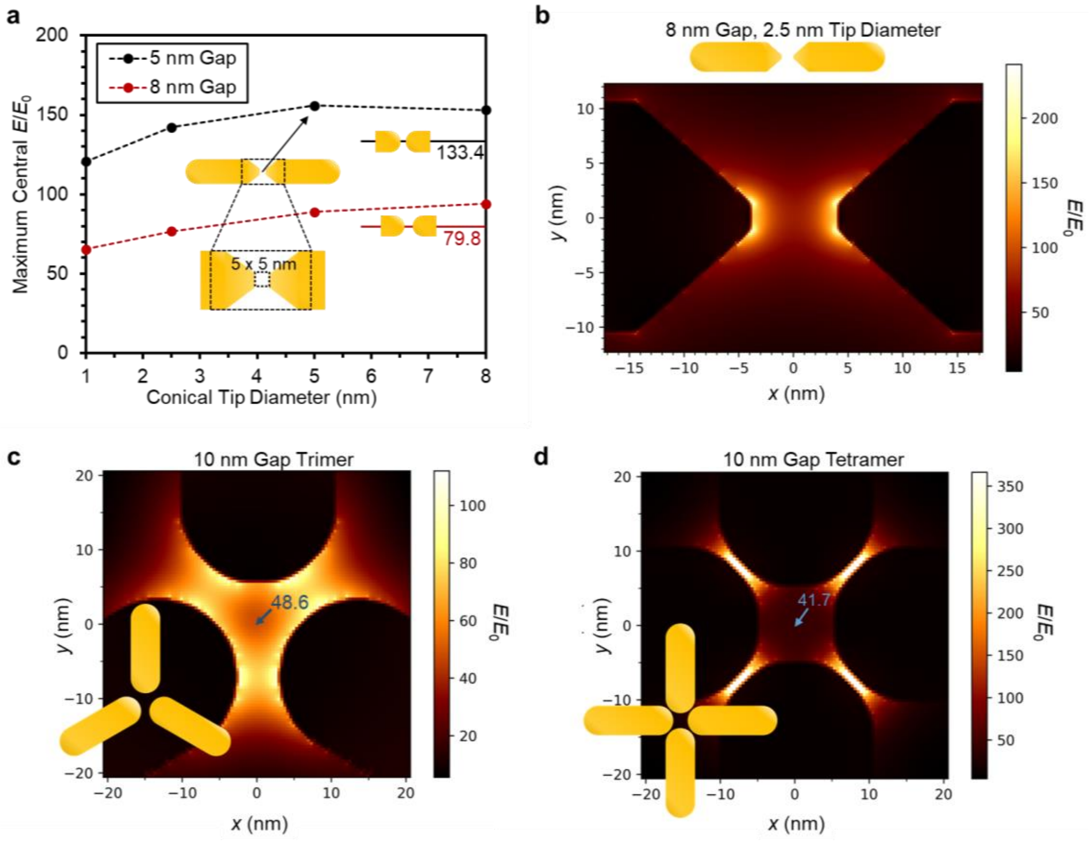

Supplementary Figure 16: Gold nanorod dimers compared to different geometries. a Comparison between the calculated maximum central EM field enhancement of spherically and conically (sharpness defined by truncation/tip diameter) shaped gold nanorod dimers tips. **b** Calculated map of the maximum *E*-field enhancement (at 763 nm) for two conically capped nanorods (2.5 nm tip diameter) separated by 8 nm. **c** Map of the maximum *E*-field enhancement (at 807 nm) of a gold nanorod trimer for illumination with circularly polarized light. The nanorods were aligned around a central spot with a diameter of 10 nm. **d** Map of the maximum *E*-field enhancement (at 866 nm) for a gold nanorod tetramer for illumination with circularly polarized light. Again, nanorods were aligned around a central spot with a diameter of 10 nm. The number insets in C and D indicate the maximum *E*-field enhancement in the center. 64x21 nm gold nanorods were assumed for all calculations.

Supplementary References

1. Ghosh A, *et al.* Graphene-based metal-induced energy transfer for sub-nanometre optical localization. *Nat Photonics* **13**, 860-865 (2019).

2. Wu TC, Vasudev M, Dutta M, Stroscio MA. Raman and Surface-Enhanced Raman Scattering (SERS) Studies of the Thrombin-Binding Aptamer. *Ieee T Nanobiosci* **12**, 93-97 (2013).
